# ReMixT: clone-specific genomic structure estimation in cancer

**DOI:** 10.1186/s13059-017-1267-2

**Published:** 2017-07-27

**Authors:** Andrew W. McPherson, Andrew Roth, Gavin Ha, Cedric Chauve, Adi Steif, Camila P. E. de Souza, Peter Eirew, Alexandre Bouchard-Côté, Sam Aparicio, S. Cenk Sahinalp, Sohrab P. Shah

**Affiliations:** 1 Department of Molecular Oncology, BC Cancer Agency, 675 West 10th Avenue, Vancouver, BC Canada; 2 Department of Pathology and Laboratory Medicine, University of British Columbia, 2329 West Mall, Vancouver, BC Canada; 3 Department of Statistics, Oxford University, 24-29 St Giles, Oxford, United Kingdom; 4 Ludwig Institute for Cancer Research, Oxford University, Old Road Campus Research Building, Headington, Oxford, United Kingdom; 5 Dana-Farber Cancer Institute, 450 Brookline Ave, Oxford, Boston USA; 6 Eli and Edythe L. Broad Institute of MIT and Harvard, 415 Main Street, Cambridge, MA USA; 7 Department of Mathematics, Simon Fraser University, 8888 University Drive, Burnaby, BC Canada; 8 Department of Statistics, University of British Columbia, 2329 West Mall, Vancouver, BC Canada; 9 Vancouver Prostate Centre, 2660 Oak Street, Vancouver, Canada; 10 Department of Computer Science, Indiana University Bloomington, 107 S. Indiana Avenue, Bloomington, IN USA

**Keywords:** Cancer genomics, DNA sequencing, Tumour heterogeneity, Genomic rearrangement, Copy number variation

## Abstract

Somatic evolution of malignant cells produces tumors composed of multiple clonal populations, distinguished in part by rearrangements and copy number changes affecting chromosomal segments. Whole genome sequencing mixes the signals of sampled populations, diluting the signals of clone-specific aberrations, and complicating estimation of clone-specific genotypes. We introduce ReMixT, a method to unmix tumor and contaminating normal signals and jointly predict mixture proportions, clone-specific segment copy number, and clone specificity of breakpoints. ReMixT is free, open-source software and is available at http://bitbucket.org/dranew/remixt.

**Electronic supplementary material:**

The online version of this article (doi:10.1186/s13059-017-1267-2) contains supplementary material, which is available to authorized users.

## Background

Chromosomal rearrangements pattern the genomes of cancer cells. Owing to various forms of DNA repair deficiency, such structural variations accumulate on cell division, leading to genome instability in the life histories of cancer cells. Coupled with evolutionary selection and clonal expansion, genomic instability and consequent segmental aneuploidies mark expanded cell populations within a tumour, forming important components of their genotypes. Within each tumour, branched evolution produces mixed populations of tumour cells with ancestrally related, but divergent chromosomal structures.

Accurate detection and quantification of genomic structural changes in a population of cancer cells as measured by bulk, whole genome sequencing (WGS) remains a significant computational challenge. The process of DNA extraction from a tumour sample pools and admixes molecules from the input material without labelling the assignment of DNA to its parent cell. The resulting sequencing data represent a randomly sampled subset of DNA fragments from the admixed pool, leaving the problem of unmixing the structural rearrangements which mark the constituent clones in the input material. The key difficulty of the problem is that the admixed pool dilutes the signal of genomic rearrangements and copy number alterations in the data, often to a level approaching that of the experimental noise.

Rearrangements and copy number changes are intrinsically linked, with unbalanced rearrangements producing changes in copy number, and loss or gain of rearranged chromosomes resulting in segment-specific copy changes. Rearrangement breakpoints representing tumour-specific adjacencies can be predicted with reasonable accuracy from WGS data using a variety of tools [1–4]. However, existing methods for copy number analysis do not consider tumour-specific adjacencies, and instead model segments as adjacent only if they are adjacent in the reference genome [5–9]. This results in only partial ability to leverage the spatially correlated nature of the data to borrow statistical strength.

We propose that breakpoints provide the potential for a more comprehensive model of genome structure. Knowledge of long-range connectivity between segments of a cancer genome provides the opportunity to simultaneously analyse breakpoints and copy number in a unified model and to reconstruct the true genomic topology. Integrating both copy number and breakpoints also provides additional information about each breakpoint: whether the breakpoint is real or a false positive, the prevalence of the breakpoint in the clone mixture, and the number of chromosomes harbouring the breakpoint per clone. A natural hypothesis then emerges: a comprehensive model of genome structure will improve both copy number inference and biological interpretation through reconstructed tumour genomes.

Some progress has been made on more comprehensive modelling of genome structure in tumour clones. Mahmoody et al. [10] propose an algorithm to infer missing adjacencies in a mixture of rearranged tumour genomes; however, they do not model copy number. Zerbino et al. [11] propose a framework for sampling from the rearrangement history of tumour genomes. Oesper et al. [12] propose PREGO, a method for inferring the copy number of segments and breakpoints using a genome graph-based approach, though they do not model normal contamination or tumour heterogeneity, limiting applicability of their method to real tumour data. More recently, Li et al. [13] formulate a Markov random field model of allele-specific copy number change and apply their method, Weaver, to samples harbouring a single tumour clone and contaminating normal cells.

We propose ReMixT, a method for jointly inferring clone mixture proportions, clone- and allele-specific segment copy numbers, and clone-specific breakpoint copy number from WGS data. We formulate the problem as a posterior inference problem on a probabilistic graphical model. Our model captures the spatial correlation both between segments that are adjacent in the reference genome in addition to correlations between segments adjacent in the tumour genome as nominated by predicted breakpoints. We describe an algorithmic solution using structured variational inference. Importantly, our algorithm is similar in complexity to a breakpoint-naive hidden Markov model (HMM) of segment copy number. We leverage haplotype blocks to more accurately measure allele-specific read counts and infer allele-specific copy number for each clone.

We assert that joint inference of all three features of genome sequencing described above will result in more accurate prediction compared to independent inference. Knowledge of rearrangement breakpoints will prevent the smoothing over of copy number changes produced by true rearrangements. Incorrect smoothing of highly rearranged chromosomes may have detrimental effects on the estimation of mixing proportions and variance parameters, as the model would be forced to compensate for an unexpected increase or decrease in read depth across the smoothed chromosomes. Finally, post hoc prediction of rearrangement breakpoint copy number based on segment copy number may fail if the exact locations of associated copy number transitions are not identified, particularly for rearrangements present in a minor fraction of clones.

We show using simulations that a more complete model of genome structure that includes breakpoint information results in improved inference of mixture proportion and segment copy number over an otherwise equivalent HMM combined with post hoc annotation. Performance improvements are most dramatic when the proportion of one clone is small. We benchmark ReMixT against TITAN [5], THetA2 [14], Battenberg [8], and CloneHD [7] using a novel framework for generating realistic partially simulated WGS datasets from an existing WGS dataset. As further validation, we applied ReMixT to four primary tumour samples from a patient with high-grade serous ovarian cancer (HGSOvCa) and performed single cell breakpoint sequencing on a subset of the clone-specific breakpoints. Next we applied ReMixT to a primary breast cancer sample and its derived mouse xenograft samples, recapitulating previously described [15] clonal dynamics identified using deep sequencing of single nucleotide variants (SNVs). Finally, we analysed two HGSOvCa cell lines, providing examples of how ReMixT-predicted clone-specific breakpoints can phase disparate subclonal genomic regions into partial tumour chromosomes towards fully reconstructing clone-specific cancer genomes.

## Results

### The ReMixT model of genome structure

We consider the problem of predicting segment and breakpoint copy number given WGS data from tumour and matched normal samples. Assume as input a set of alignments of uniquely mapped concordant reads and a set of putative breakpoints predicted from discordant reads. Given *N* segments indexed by *n*, *n*∈{1…*N*}; *K* breakpoints indexed by *k*, *k*∈{1…*K*}; and assuming *M* clones indexed by *m*, *m*∈{1…*M*}, we aim to predict the following:

1. Mixture proportions of tumour clones and normal cells *ρ*_*m*_
2. Clone- and allele-specific copy numbers of genomic segments *c*_*nm*_
3. Clone-specific copy number of rearrangement breakpoints *b*_*km*_

#### Data preprocessing

Preprocessing of tumour WGS data produces measured total and allele-specific read counts for a set of genomic segments in addition to tumour-specific adjacencies between those segments. First, the genome is partitioned into regular length segments, with segments containing the breakends of input breakpoints further partitioned such that each breakend coincides with a segment boundary. Total read counts are obtained by counting the number of uniquely aligned paired-end reads fully contained within each segment. Next, haplotype blocks are predicted from single nucleotide polymorphisms (SNPs) using shapeit2 [16] and a 1000 Genomes reference panel. Reads containing heterozygous SNPs are assigned to haplotype blocks, and haplotype block counts are aggregated within segments, resulting in per-segment allele-specific read counts. GC and mappability biases contribute significant variance to segment read counts. We use a position-specific model [17] to calculate a bias-adjusted *effective length* for each segment, where segments with shorter effective lengths are statistically less well represented by read counts. For visualization purposes, we calculate *raw* major and minor copy numbers for each segment from observed depths and allele ratios and inferred normal and tumour depth. Additional details are provided in Additional file 1: Sections 1.1 and 1.2.

#### Probabilistic model

**Fig. 1:**
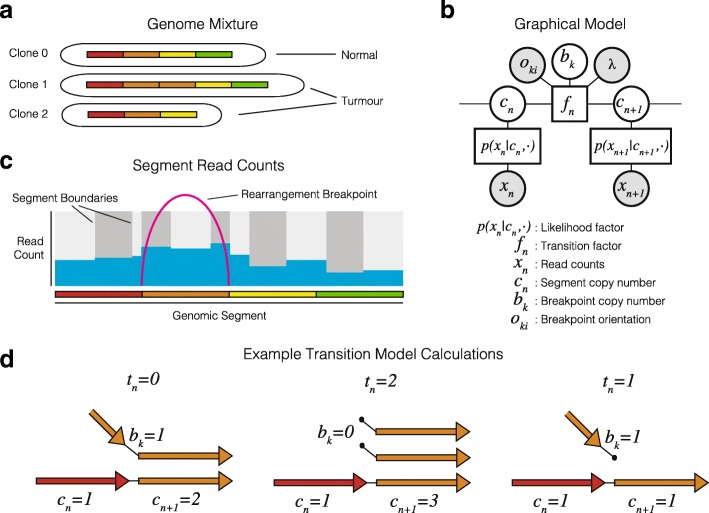
An overview of the ReMixT Method. **a**) Bulk sequencing is applied to a mixture of cells modeled as a set of clones of unknown proportion each with distinct sets of chromosomes with unknown structure. **b**) Observed data include binned read counts per segment, and rearrangement breakpoints connecting segment ends. **c**) The ReMixT graphical model as a factor graph. **d**) Calculation of the transition factor involves calculating the number of *telomeres* *t*, the number of segment ends left unconnected to another segment end in the model

We propose a probabilistic model of genome structure and a structured variational inference algorithm for calculating the optimal clone mixture and segment and breakpoint copy number (Fig. 1). Below we focus on a model of total copy number and defer the details of the allele-specific model and modelling of outliers to Additional file 1: Section 1.3. Let *p*(*x*|*c,h,l*,*θ*) be the likelihood of observed total read count *x* given per clone segment copy number *c*, segment length *l*, global likelihood parameters *θ*, and per clone haploid read depths *h*. The haploid read depths encode both the mixture and depth of sequencing and are specified as reads per nucleotide for a single copy of a segment. The expected read count *μ*_*n*_ of segment *n* is a linear combination of the segment length, clone-specific copy number, and clone-specific haploid read depth, summed over clones (Eq. 1):

1 $$\begin{array}{@{}rcl@{}} \mu_{n} = l_{n} \sum_{m} h_{m} c_{nm}  \end{array} $$

A reasonable starting point is to assume read counts are Poisson distributed [18] (*x*_*n*_∼ Pois(*μ*_*n*_)); however, we show in Additional file 1: Section 1.2.3, that a two-component negative binomial mixture provides a significantly better fit to real data.

**Fig. 2:**
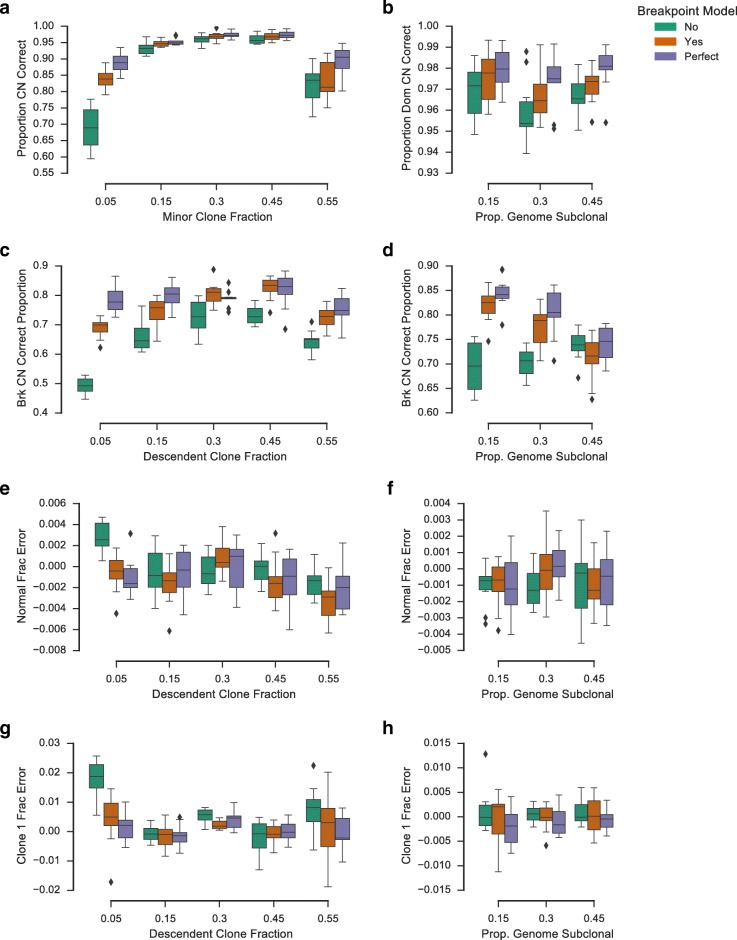
Simulation results for the integrated breakpoint model and an equivalent hidden Markov model (*HMM*) with postprocessing to infer breakpoint copy number. Also shown are results for the breakpoint model with perfect initialization. Two sets of simulations were performed, varying fraction of the descendant tumour clone (*left column*) and proportion of the genome with divergent copy number (*right column*). Boxplots show proportion of the genome (**a**, **b**) and proportion of breakpoints (**c**, **d**) for which the tool correctly called clone-specific copy number, in addition to relative normal fraction error (**e**, **f**) and relative minor clone fraction error (**g**, **h**). *Boxes* show the interquartile (*IQR*) range with a *line* depicting the median. Whiskers extend 1.5×IQR above quartile 3 and below quartile 1. *Diamonds* show positions of outlier data points

Let *p*(*C,B*|*O*,*λ*) be the joint probability of segment and breakpoint copy number (*C* and *B* respectively) given breakend orientations *O*. We assume the copy numbers of a sequence of segments have the Markov property-given breakpoint copy number, and represent the resulting chain structure as a product of un-normalized transition factors^1^. A breakpoint with breakend interposed between two segments will result in a copy number transition between those segments. For instance, a transition in copy number is expected between two segments to either side of the start of a deletion, with the difference in segment copy number equal to the number of chromosomes harbouring the deletion event, or equivalently, the number of copies of the deletion breakpoint. A mismatch in segment and breakpoint copy number implies that at least one segment end is left disconnected (Fig. 2 d). We call these free ends *telomeres*, and define the transition factors of our probability model in terms of the number of telomeres *t* implied by the segment and breakpoint copy number. Without a breakpoint, the number of telomeres is simply the absolute difference in copy number between adjacent segments *t*(*c,c* ^′^)=|*c*−*c* ^′^|. Depending on its orientation, a positive copy number for a breakpoint may explain some or all of the difference in copy number between adjacent segments. The number of telomeres at a transition coincident with a breakpoint can thus be calculated as *t*(*c,c* ^′^,*b* ^′^,*o*)=|*c*−*c* ^′^−*o*·*b*|, with orientation *o*∈{−1,+1}. For multiple clones, *t* may be a more complex function of the copy number differences for each clone (see Additional file 1: Section 1.4).

Define transition factors $f(c, c^{\prime }, b | o, \lambda) = e^{-\lambda t(c, c^{\prime }, b | o)}\phantom {\dot {i}\!}$, and let *k*_*n*_ be the index of the breakpoint interposed between segment *n* and *n*+1. Write the joint probability over the observed read counts and segment and breakpoint copy number as given by Eq. 2:

2 $$\begin{array}{@{}rcl@{}} {}p(X, C, B | h, L, O, \theta, \lambda) &=& p(X | C, L, h, \theta) p(C, B| O, \lambda)  \\ &\propto& \prod_{n=1}^{N} p(x_{n} | c_{n}, h, l_{n}, \theta) \\ &&\times\prod_{n=1}^{N-1} f(c_{n}, c_{n+1}, b_{k_{n}} | o_{n}, \lambda)  \\  \end{array} $$

Exact inference in the ReMixT model is intractable due to additional dependencies introduced by modelling the long-range connectivity of breakpoints.

#### Structured variational inference

We are seeking to infer the posterior probability *p*(*z*|*x*) of the unobserved model variables *z* given observed data *x*. The variational inference approach seeks to approximate an intractable posterior *p*(*z*|*x*) with a more tractable family of distributions *q*(*z*), typically characterized by an increased number of parameters and fewer dependencies [19]. An optimal *q*(*z*) is computed by minimizing the Kullback-Leibler (KL) divergence between *p*(*z*|*x*) and *q*(*z*) as given by Eq. 3:

3 $$\begin{array}{@{}rcl@{}} {}D_{\operatorname{KL}} \left(q(z) | p(z | x) \right) &=& \int q(z) \log \left(\frac{q(z)}{p(z|x)} \right) dz  \\ &=& \log p(x) - \int q(z) p(x, z) dz \\ &&+ \int q(z) \log q(z) dz  \\ &=& \log p(x) - \mathbb{E}_{q} \left[ p(x, z) - \log q(z) \right]  \end{array} $$

The expectation given in the final form of Eq. 3 forms a lower bound on the model evidence *p*(*x*), since *D*_KL_(*q*(*z*)|*p*(*z*|*x*)) is positive and approaches zero for a perfect approximation. Importantly, the difficult problem of directly minimizing the KL divergence is equivalent to the easier problem of maximizing this evidence lower bound (ELBO). The mean field approximation assumes a distribution $q(z) = \prod _{i} q_{i}(z_{i})$ that factorizes over single model variables. In structured variational inference, each *z*_*i*_ is a disjoint set of model variables, allowing *q* to have a more complex dependency structure that better approximates the posterior [20, 21]. Independence between factors of *q* allows for application of a coordinate descent algorithm that iteratively maximizes the ELBO with respect to each *q*_*i*_ using general updates given by Eq. 4:

4 $$\begin{array}{@{}rcl@{}} \log q^{*}(z_{j}) &=& \mathbb{E}_{\prod_{j \neq i} q_{j}(z_{j})}[\log p(x, z)] + \operatorname{const}  \end{array} $$

We approximate the posterior *p*(*C,B,h*,*θ*|*X,L,O*,*λ*) using a distribution *q* with factorization given by Eq. 5:

5 $$\begin{array}{@{}rcl@{}} q(C, B, h, \theta) &=& q(h) q(\theta) q(C) \prod_{k} q_{k}(b_{k})  \end{array} $$

Taking a variational expectation maximization (EM) approach, we specify the distributional form of *q*(*h*) and *q*(*θ*) to be the Dirac delta function, and compute point estimates for those parameters. Applying Eq. 4 to *q*(*C*) results in Eq. 6 ^2^:

6 $$\begin{array}{@{}rcl@{}} \log q^{*}(C) &=& \sum_{B} \left(\prod_{k} q(b_{k}) \right) \log p(X, C, B, h, \theta | L, O, \lambda) \\ &&+ \operatorname{const}  \\ &=& \sum_{n} \zeta_{n}(c_{n}) + \sum_{n=1}^{N-1} \zeta_{n}(c_{n}, c_{n+1}) + \operatorname{const}  \end{array} $$

7 $$\begin{array}{@{}rcl@{}} \zeta_{n}(c_{n}) &=& \log p(x_{n} | c_{n}, h, l_{n}, \theta)  \end{array} $$

8 $$\begin{array}{@{}rcl@{}} \zeta_{n}(c_{n}, c_{n+1}) &=& \sum_{b} q_{k_{n}}(b) \log f(c_{n}, c_{n+1}, b | o_{n}, \lambda)  \end{array} $$

By inspection, the probability distribution *q* ^∗^(*C*) given by Eq. 6 has a chain topology equivalent to an HMM, with an emission calculated as a function of the read count likelihood and transition matrices calculated by modifying *f* according to $q_{k_{n}}(b)$ (Eqs. 7 and 8). The emission and transition terms *ζ*_*n*_(*c*_*n*_) and *ζ*_*n*_(*c*_*n*_,*c*_*n*+1_) define the variational parameters of *q*(*C*). The sum product algorithm can be used to calculate the single and pairwise posterior marginal probabilities of *q*(*C*), denoted *γ*_*n*_(*c*) and *γ*_*n*_(*c,c* ^′^) respectively. The posterior marginals of *q*(*C*) will appear in the updates of the other factors of *q*, as shown below.

Applying Eq. 4 to optimize *q*_*k*_(*b*_*k*_) results in Eq. 9:

9 $$\begin{array}{@{}rcl@{}} \log q_{k}^{*}(b_{k}) &=& \sum_{C} q(C) \log p(X, C, B, h, \theta | L, O, \lambda) + \operatorname{const}  \\ &=& \sum_{n: k_{n}=k} \sum_{c} \sum_{c'} \gamma_{n}(c, c') \log f(c, c', b_{k} | o, \lambda) \\ &&+ \operatorname{const}  \end{array} $$

Intuitively, the variational updates for *q*(*C*) and *q*_*k*_(*b*_*k*_) described above involve first updating the transition matrices of an HMM, weighting specific transitions that correspond to copy number changes induced by high-probability breakpoint copy number states, and then updating breakpoint copy number states according to the probabilities over adjacent segments in the HMM.

Since the entropy of a delta function is constant, optimal estimates of *h* and *θ* involve minimizing only the $\mathbb {E}_{q} \left [ \log p(x,z) \right ]$ term of the ELBO. Read counts are independent of breakpoints given segment copy number; thus, the expectation is calculated over *q*(*C*) only (Eq. 10). Minimization is accomplished by computing derivatives with respect to the parameters and using quasi-Newton methods to find a local minimum.

10 $$\begin{array}{@{}rcl@{}} {}\mathbb{E}_{q} \left[ \log p(x,z) \right] &=& \sum_{C} q(C) \log p(X, C, B, h, \theta | L, O, \lambda)  \\ &=& \sum_{n} \sum_{c} \gamma_{n}(c) \log p(x_{n} | c, h, l_{n}, \theta)  \end{array} $$

### Realistic simulations of bulk genome sequencing

We developed a principled method of simulating rearranged genomes that fulfilled three important criteria. First, the simulated tumour genomes were required to have been produced by a known evolutionary history composed of duplication, deletion, and balanced rearrangement events applied successively to an initially non-rearranged normal genome. Second, the copy number profile of the simulated tumour genome should be reasonably similar to those of previously observed tumours. Third, the simulated data should be subject to the same biases seen in real genome sequence data.

To satisfy the first two criteria, we developed a sampling framework for generating realistic evolutionary histories based on a scoring and re-sampling strategy (see Additional file 1: Section 2.1). This first step produces a set of rearrangements, in addition to per-clone per-segment copy numbers. WGS read-level data are generated from segment copy numbers in one of two possible ways. For *segment count simulations*, read counts are simulated directly from a likelihood model given simulated segment copy number. For *aligned read re-sampling*, individual reads are re-sampled from a very high depth *source* normal genome dataset based on simulated segment copy number. By using an appropriate likelihood model, segment count simulations can be used to generate read counts with a distribution that reflects the over-dispersion and outliers in real data. Aligned read re-sampling datasets are computationally more intensive to generate, but are able to produce read count data with GC and mappability bias similar to that of the source dataset. See Additional file 1: Section 2.2 for additional details.

### Breakpoint model improves inference for segment count simulations

We first sought to understand the benefit of an integrated breakpoint model using segment count simulations. We compared the ReMixT model with an equivalent breakpoint-naive HMM followed by post hoc breakpoint copy number calculation. For the breakpoint-naive model, we first infer segment copy number using the ReMixT model with breakpoint copy number at zero. We then use a simple greedy algorithm (see Additional file 1: Section 2.5) to perform a post hoc computation of the breakpoint copy number based on the segment copy number inferred using the HMM. As variational inference is sensitive to initialization, we also included results using the ReMixT breakpoint model with perfect initialization. We performed our evaluation on two sets of simulations, one in which we varied the proportion of the genome simulated to be subclonal, and one in which we varied the descendant clone fraction (see Additional file 1: Section 2.3 for details)^3^.

We evaluated the breakpoint model and the HMM on the model’s ability to recover the true clonal mixture, segment copy number, and breakpoint copy number (Fig. 2). Mixture prediction was assessed by calculating the relative deviation of the predicted normal fraction and descendant clone fraction from the simulated values. Segment and breakpoint copy number prediction was assessed by calculating the proportion of segments/breakpoints for which the true clone-specific copy number was recovered by the method.

**Fig. 3:**
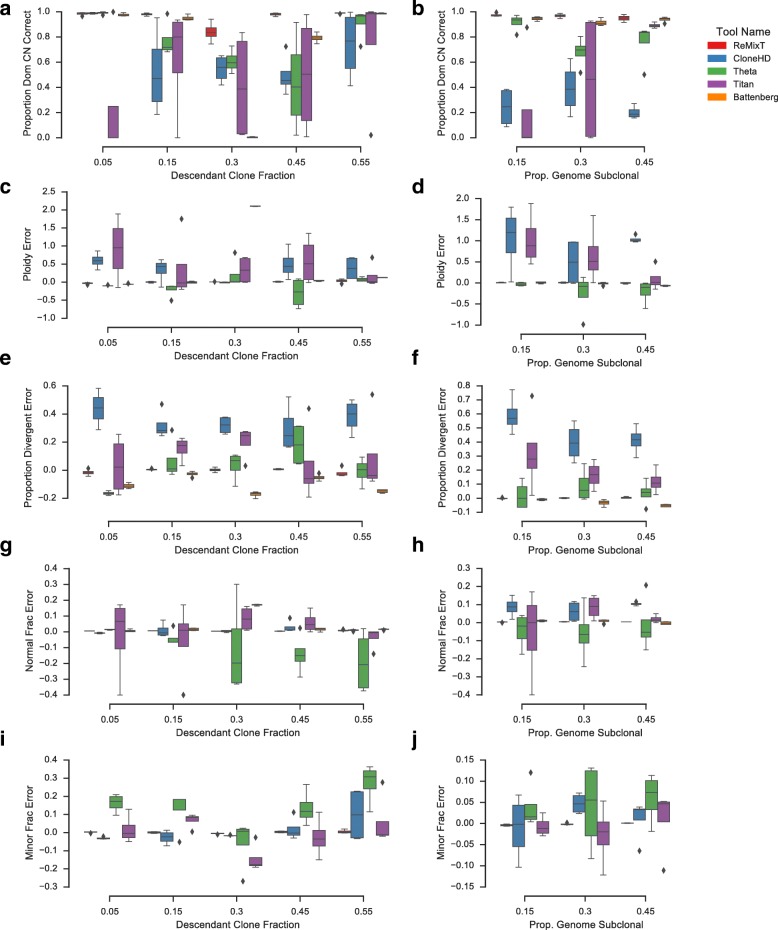
Performance comparison of ReMixT with CloneHD, TITAN, Battenberg, and THetA using read re-sampling simulations. Two sets of simulations were performed, varying fraction of the descendant tumour clone (*left column*) and proportion of the genome with divergent copy number (*right column*). *Boxplots* show proportion of the genome for which the tool correctly called the copy number of the dominant clone (**a**, **b**), relative mean ploidy error compared to simulated (**c**, **d**), relative proportion divergent error compared to simulated (**e**, **f**), relative normal fraction estimation error compared to simulated (**g**, **h**), and relative minor clone fraction estimation error compared to simulated (**i**, **j**). Battenberg was excluded from the minor clone fraction benchmark, as it does not produce a global estimate of this parameter. *Boxes* show the interquartile (*IQR*) range with a *line* depicting the median. Whiskers extend 1.5×IQR above quartile 3 and below quartile 1. *Diamonds* show positions of outlier data points

For both segment and breakpoint copy number prediction, the breakpoint model outperformed the baseline HMM. The proportion of segment copy number called correctly was significantly higher for the breakpoint model for all simulations with the exception of those simulations with a descendant clone fraction of 55% (paired *t* test, *p* value <0.05, Fig. 3 a and b). Additionally, the proportion of breakpoints with correctly predicted copy number was significantly higher for the breakpoint model for all simulations with the exception of those with the proportion of the genome subclonal set at 45% (paired *t* test, *p* value <0.05, Fig. 3 c and d). Improvement with respect to prediction of minor clone fraction was observed for descendant clone fractions 0.05 and 0.3 (paired *t* test, *p* value <0.05, Fig. 3 g). No improvement was observed with respect to normal fraction prediction, though we did observe a decrease in accuracy for descendant clone fraction 0.55 (paired *t* test, *p* value =0.03, Fig. 3 e). Perfect initialization showed improved results over our current initialization method, indicating additional room for improvement with respect to this aspect of the algorithm.

### Comparison with existing copy number inference methods

We used our aligned read re-sampling framework to compare the performance of ReMixT to four existing methods for subclonal copy number inference: TITAN [5], CloneHD [7], Battenberg [8], and THetA2 [12, 14]. We performed our comparison on two sets of genome mixtures, one in which we varied the proportion of the genome simulated to be subclonal, and one in which we varied the descendant clone fraction. We used aligned read re-sampling to produce realistic simulated datasets using 200X sequencing of the NA12878 hapmap individual provided by Illumina [22]. Each tool was run with default parameters according to available instructions (see Additional file 1: Section 4 for details).

Performance of the four tools varied significantly across each measure (Fig. 3). CloneHD was unable to recover the copy number of the dominant clone with reasonable accuracy for a majority of the simulations (< 43% accurate for 50% of simulations). In general, CloneHD copy number results showed a higher mean ploidy and higher divergent proportion (proportion of the genome predicted to have clonally divergent copy number) than simulated results (average 37% higher and 44% higher respectively). However, in many instances, CloneHD was able to estimate normal fraction with reasonable accuracy (within 6.6% of simulated for 50% of the simulations). Minor clone fraction estimation was less accurate (within 28% of simulated for 50% of the simulations). Our results imply that CloneHD is prone to over-fitting, producing unrealistic copy number profiles.

THetA, by contrast, produced solutions accurate with respect to mean ploidy (within 6.5% of simulated for 75% of simulations) and, to a lesser extent, divergent proportion (within 20% of simulated for only 25% of simulations). Additionally, THetA copy number predictions were more consistent in their accuracy, with the dominant copy number predicted with greater than 81% accuracy for 50% of the simulations. The normal fraction estimation error was in general higher than for the other tools (within 17% of simulated for 50% of simulations). THetA’s estimated descendant clone fractions were also less accurate than those of the other tools (within 21% of simulated for only 25% of simulations).

TITAN’s results were the most variable, with dominant copy predicted accurately for a large number of simulations (> 88% for 25% of simulations) but poorly for many other simulations (< 21% for 25% of simulations). As with CloneHD, TITAN appeared to over-fit for a subset of the simulations, producing solutions for which mean ploidy and divergent proportion were higher than simulated (> 28% higher than simulated ploidy for 25% of simulations and > 66% higher than simulated divergent proportion for 50% of simulations). TITAN estimated normal fractions with low error for a majority of simulations (within 5% of simulated for 50% of simulations), though prediction of minor clone fractions was more variable (error greater than 19% of simulated for 75% of simulations).

Battenberg’s results were the most consistent of the competing tools. For the simulations with 50/50 tumour mixtures, Battenberg produced a solution at double the simulated ploidy, highlighting the unidentifiability of this particular scenario. Excluding the 50/50 tumour mixture simulations, Battenberg predicted dominant copy number within 3% for 75% of the simulations and ploidy within 4% for 75% of the simulations. Battenberg in general under-estimated the divergent proportion, 13% lower than simulated for 75% of simulations. Normal fractions were also accurate, within 6% of simulated for 100% of simulations, excluding 50/50 mixtures. Battenberg does not estimate minor clone fraction and was thus excluded from such analyses.

ReMixT consistently outperformed the four competing tools on all measures. For 75% of the simulations, ReMixT was able to infer integer copy number for both clones with greater than 91% accuracy. Lower accuracy results were obtained for 50/50 tumour mixtures, primarily due to the inherent ambiguity of assigning copy numbers to specific clones for such mixtures. Normal fraction estimation was slightly biased, and was over-estimated by 1.4% of simulated on average, though never by more than 2.6%. As expected, minor clone fraction estimation was less accurate for mixtures with the smallest simulated minor clone fractions, up to 50% of simulated, averaging 5%. For the remaining simulations minor clone fraction estimation error averaged 0.6% with a maximum of 8%.

### Targeted single cell validation of clone-specific breakpoints

Next we sought to establish the accuracy of breakpoint copy number inference in a realistic setting using targeted single cell sequencing in a set of specially separated high-grade serous ovarian tumour samples [23]. The set of samples included two obtained from the patient’s right ovary, one from the left ovary, and one from the omentum (Fig. 5 b). Each sample was whole genome sequenced to an approximate depth of 30X.

**Fig. 4:**
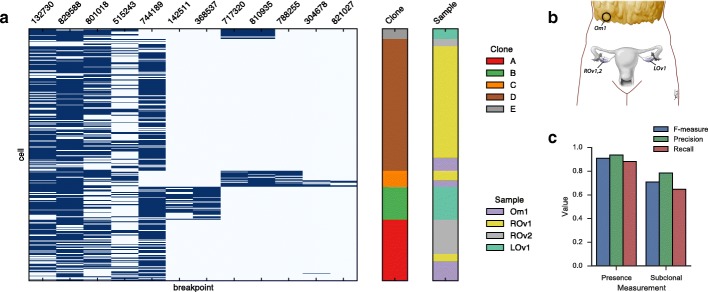
Single cell validation of ReMixT results for 12 breakpoints in 294 cells from 4 HGS Ovarian tumour samples: Omentum 1 (Om1), Right Ovary 1 and 2 (ROv1 and ROv2), and Left Ovary 1 (LOv1). (**a**) Breakpoint (*x*-axis) by cell (*y*-axis) presence (*dark blue*) / absence (*light blue*) with cells annotated by sample of origin and clone as inferred by the Single Cell Genotyper. (**b**) Approximate anatomic location of the 4 tumour samples. (**c**) F-measure, precision and recall for ReMixT calls of breakpoint presence and subclonality

We hand-selected 12 breakpoints associated with putative copy number changes for validation by targeted single cell sequencing (Fig. 4). Specifically, for each of the 12 candidate breakpoints, at least one breakend coincided with a transition in copy number in at least one sample, where copy number was inferred using an earlier version of ReMixT [23]. In addition, we selected 60 somatic and 24 germline single nucleotide changes based on their utility as clonal markers [23]. Targeted single cell sequencing was performed as previously described [23], cells were clustered into clones using the Single Cell Genotyper [24], and breakpoints were assigned to clones if they were present in at least three cells of that clone. Joint analysis of the breakpoint and single nucleotide data produced a robust estimate of the clonal genotypes with respect to the targeted breakpoints (Fig. 4 a).

Next we evaluated the ability of ReMixT to accurately determine which breakpoints were present/absent and clonal/subclonal in each sample. We calculated the *F* measure for present/absent and clonal/subclonal calls (Fig. 4 c). *F* measure values were similar to results obtained from running ReMixT on aligned read re-sampling simulations.

### Tracking clonal expansions using clone-specific breakpoints

**Fig. 5:**
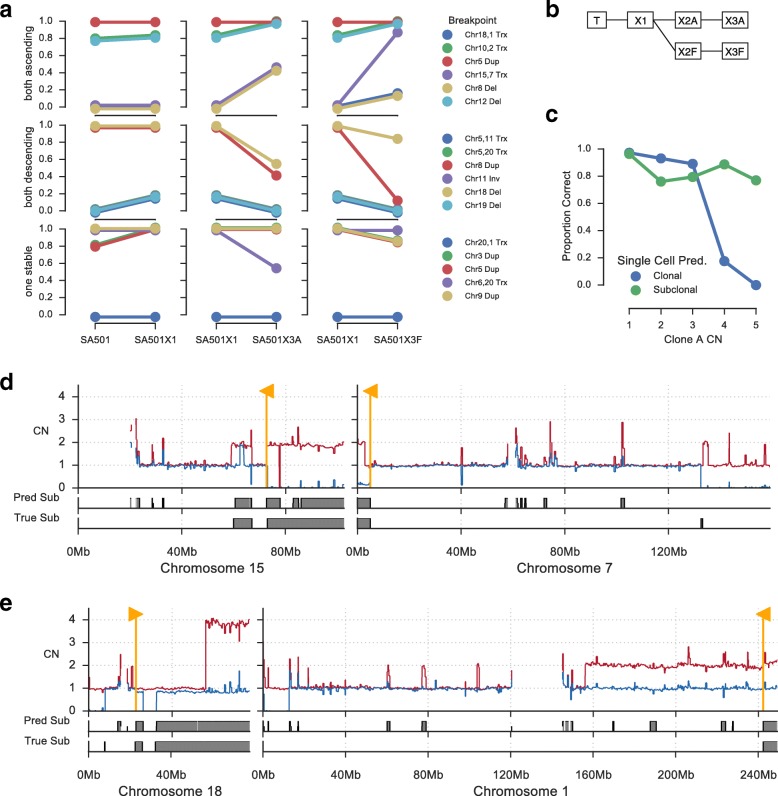
Tracking clonal expansions in xenograft passages. **a** Breakpoints identified by ReMixT as clone-specific were classified according to their clonal prevalence change between SA501X1A and replicate xenograft passages SA501X3A and SA501X3F. All breakpoints could be classified as ascending in both SA501X3A and SA501X3F, descending in both, or stable in at least one. Shown are the clonal prevalence changes between pairs of samples for which WGS was available. **b** Relationship between primary tumour sample T and xenograft passages X*. **c** Accuracy of copy number inference for X3F based on single cell whole genome sequencing. Shown is the proportion of regions with correctly predicted copy number (*y*-axis) for each clone A copy number (*x*-axis), split between clonal and subclonal (*blue/green*) as determined from single cell data. **d** Copy number profile (*top*) for chromosomes 7 and 15 showing corroboration between single cell (*bottom*) and ReMixT (*middle*) subclonal copy number prediction. *Yellow flags* show the location of translocation breakpoints predicted to be subclonal by ReMixT. **e** Similarly, chromosomes 1/18 translocation breakpoints predicted to be subclonal by ReMixT. Copy number plots show raw major (*red*) and minor (*blue*) copy numbers

Several previous studies have used clone-specific SNVs to identify patterns of clonal evolution [25], infer patterns of cancer cell dissemination to metastatic sites [23, 26], and track expansion and contraction of tumour clones over time and in response to therapy [27] and in response to xenograft passaging [15]. We sought to evaluate the utility of clone-specific breakpoints predicted by ReMixT for investigating clonal evolution in successive xenograft passages. To this end, we analysed primary and xenograft tumour samples derived from a patient with breast cancer (SA501 from [15]). Our analysis focused on four samples, the primary tumour sample and three xenograft samples labelled X1A, X3A, and X3F. The relationship between these four samples and the additional two un-sequenced xenograft samples X2A and X2F is shown in Fig. 5 b.

For validation of X3F clone-specific copy number changes, we used recently published single cell WGS data [28]. We inferred total integer copy number and performed phylogenetic analysis using previously described techniques [15, 28]. Three major clones were identified. Proportions of cells assigned to each clone were 0.82, 0.11, and 0.07 for clones A, B, and C respectively. Clones B and C were highly similar and formed a distinct clade; thus, for this analysis we merged clones B and C. For clone A and merged clone BC, we reconstructed clone copy number profiles by selecting the most prevalent copy number within each clone for each segment. Segments with copy number 6 or higher were removed, as specific copy number states above 5 could not be inferred using available techniques.

ReMixT analysis using default parameters estimated a clonal mixture of 0.85 for the dominant clone and 0.15 for the minor clone. Clone-specific copy numbers matched single cell copy number for 91% of the genome. Accuracy was highest for segments in lower copy number states (≤ 3 total copies). Segments with higher copy number (≥ 4 total copies) and no clonal divergence were frequently predicted as subclonal by ReMixT, evidence that ReMixT over-fits some segments with higher copy number (Fig. 5 c). Additional disparity appeared to be the result of noisy segments in lower copy states predicted as subclonal.

Next we identified a set of high confidence subclonal breakpoints for analysis of clonal dynamics in the xenograft passages. We smoothed segments smaller than 100 kb and aggregated adjacent segments with the same allele-specific difference between clone copy numbers. We then removed segments with length less than 1 Mb or copy number greater than 4. Breakpoints were selected if they were predicted to be subclonal, and were immediately adjacent at each breakend to a segment with subclonal copy number from the above set of filtered high confidence segments. This technique was used to identify 17 subclonal breakpoints in one of X1, X3A, X3F, and X5 or the primary tumour sample. In X3F, the ReMixT copy number matched the single cell copy number for 84% of the 1-Mb regions to either side of each breakend. For 11 of the predictions, corroboration was >92%, and for the remaining predictions, corroboration was closer to 50%, indicating a lack of corroboration on one side of each breakend. Included in the set of breakpoints were inter-chromosomal translocations linking subclonal segments on disparate chromosomes, indicative of clone-specific loss or gain of rearranged tumour chromosomes (Fig. 5 d and e).

Patient SA501 was previously shown to have exhibited reproducible patterns of clonal expansions across multiple replicate xenografts using a combination of targeted bulk and single cell sequencing of SNVs [15]. In particular, X3A and X3B showed similar patterns of clonal expansions for clusters of SNVs used as clonal markers. We sought to establish whether the same clonal dynamics were evident in X3F, and whether those clonal dynamics could be understood using clonal-specific breakpoints. To that end, we classified each of the high confidence subclonal breakpoints according to whether they exhibited the same expansion patterns from X1 to X3A and X1 to X3F. Of the 17 high confidence breakpoints, 6 could be classified as ascending in both X3A and X3F, 6 as descending in both X3A and X3F, with the remaining stable from X1 to either X3A or X3F (Fig. 5 a). Strikingly, we did not identify any conflicting breakpoints, those ascending in X3A and descending in X3F or vice versa.

### Assembling tumour chromosomes using subclonal breakpoints

We applied ReMixT to WGS data from two tumour-derived cell line samples and a matched normal sample obtained from a patient with HGSOvCa [29]. The two cell lines are derived from an ascites sample (DAH354) and a primary tumour sample (DAH355) obtained during debulking surgery. Cell line samples and matched normals were sequenced to approximately 30X and analysed with ReMixT using default parameters. Tetraploid solutions were selected based on ploidy evidence from preliminary single cell sequencing experiments for DAH355 (data not shown).

As expected of HGSOvCa, the copy number profiles of the cell line samples showed substantial evidence of genome instability. For both samples, the fraction of the genome predicted to be diploid heterozygous was insignificant, and the fraction of the genome with loss of heterozygosity was 40% and 35% for DAH354 and DAH355 respectively. Both DAH354 and DAH355 showed evidence of multiple genomically distinct clonal populations, with dominant clone fractions of 0.7 and 0.61 respectively, and fraction of the diploid genome predicted as subclonal as 14% and 32% respectively. A total of 348 somatic breakpoints were identified by deStruct [4], of which 278 were determined to be present (positive copy number) by ReMixT in one or both samples. A total of 97 breakpoints were predicted to have clone-specific copy number in one or both samples, with 17 having clone-specific copy number in both samples.

**Fig. 6:**
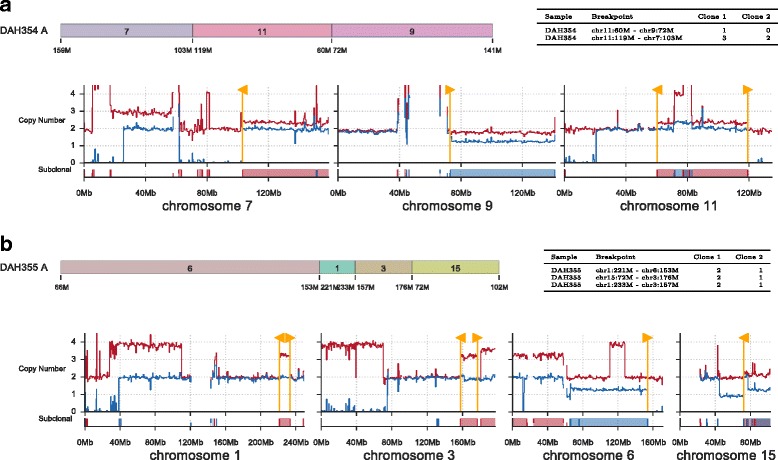
Inference of partial tumour chromosome assemblies based on linking subclonal segments and breakpoints. Two assembled chromosomes are shown for cell lines DAH354 (**a**) and DAH355 (**b**). Shown for each assembled chromosome is a schematic of the segments involved (*top left*), a table of breakpoint copy number predicted by ReMixT (*top right*), and a chromosome copy number plot (*bottom*). Each copy number plot shows raw major (*red*) and minor (*blue*) copy numbers (*top axis*), in addition to prediction of subclonality (*bottom axis*)

In both DAH354 and DAH355, we observed several clone-specific translocations adjacent to large segments with clonally divergent copy numbers. As with SA501, we suspected that the loss or duplication of a single tumour chromosome would result in multiple clonally divergent segments across the reference genome. We thus searched for clonally divergent segments connected by subclonal breakpoints as a method for understanding the structure of tumour chromosomes with divergent copy number across the clonal population (Fig. 6). In DAH354, we identified a tumour chromosome composed of three segments from reference chromosomes 7, 11, and 9 (Fig. 6 a), and in DAH355, we identified a tumour chromosome composed of four segments from reference chromosomes 6, 1, 3, and 15 (Fig. 6 b).

## Discussion

We have demonstrated that ReMixT improves both inference and interpretation of copy number changes and genomic rearrangements. Improved accuracy was observed for prediction of clone fraction, clone specific copy number, and clone specificity of breakpoints. We show how breakpoint copy number changes can be used a markers of clonal populations, and used to track clonal population dynamics in the same way as SNVs. By linking clone specific copy number changes to breakpoints we show how targeted single cell sequencing can be used to jointly profile clonal genotypes in SNV and copy number space. Furthermore, we are able to reconstruct partial tumour chromosomes lost or gained in sub-populations of cells.

Although our method shows performance gains over other methods, further improvements are possible. The performance of our variational inference algorithm is highly dependent on the quality of the initialization. Improvement may be gained using more sophisticated or informed initialization methods, or extensions to variational inference using annealing or MCMC. Our current implementation is limited to two tumour clones, largely due to the increased computational complexity of modelling additional clones. An approximating distribution factorized per clone would solve the complexity issue within the context of structured variational inference, however based on our own experimentation, such a factorization exacerbates the initialization problem and was found to be infeasible. Thus improvements to the variational inference method may also allow for the use of a more factorized approximation, removing the limitation on the number of clones.

## Conclusions

Traditionally, classes of genomic aberration have been predicted and characterized independently, with post-hoc analysis to determine correlation between events in each class. However, there are clear dependencies between classes of aberrations with respect to their generation via mutational processes and their observation using genome sequencing. A number of existing methods partially leverage class dependencies[7, 30, 31], and the development of ReMixT represents a further step towards a comprehensive model of genomic aberrations in tumour populations. We anticipate further benefit may be gained from jointly modelling copy number changes, rearrangements, SNPs and SNVs, all within the context of an appropriate phylogenetic model. Future research leveraging the patterns of genome damage and the totality of somatic alterations in a cancer’s evolutionary history to elucidate its biologic and mutagenic properties will derive benefit from ReMiXT’s improved accuracy in structural alteration detection and interpretation.

## Endnotes

^1^ A product of normalized conditional probabilities and a prior probability for the first segment would also be possible, though we believe integration of breakpoints into the model would be less intuitive.

^2^ Assuming uniform improper priors over *h* and *θ*, we have log*p*(*X,C,B*|*h*,*θ*,*L,O*,*λ*)= log*p*(*X,C,B,h*,*θ*|*L,O*,*λ*)+const.

^3^ We maintained a distinction between ancestral/descendant clone mixtures of *x* / 1−*x* and the reversed 1−*x* / *x* clone mixture, as results for these mixtures differ.

## Additional file

Additional file 1 Supplementary methods, supplementary analyses, and additional experimental results. (PDF 35000 kb)
