# Correction to: ReMixT: clone-specific genomic structure estimation in cancer

**DOI:** 10.1186/s13059-017-1327-7

**Published:** 2017-10-06

**Authors:** Andrew W. McPherson, Andrew Roth, Gavin Ha, Cedric Chauve, Adi Steif, Camila P. E. de Souza, Peter Eirew, Alexandre Bouchard-Côté, Sam Aparicio, S. Cenk Sahinalp, Sohrab P. Shah

**Affiliations:** 1 Department of Molecular Oncology, BC Cancer Agency, Vancouver, Canada; 2 Department of Pathology and Laboratory Medicine, University of British Columbia, Vancouver, Canada; 3 Department of Statistics, Oxford University, Oxford, UK; 4 Ludwig Institute for Cancer Research, Oxford University, Oxford, UK; 5 Dana-Farber Cancer Institute, Oxford, USA; 6 Eli and Edythe L. Broad Institute of MIT and Harvard, Cambridge, USA; 7 Department of Mathematics, Simon Fraser University, Burnaby, Canada; 8 Department of Statistics, University of British Columbia, Vancouver, Canada; 9 Vancouver Prostate Centre, Vancouver, Canada; 10 Department of Computer Science, Indiana University Bloomington, Bloomington, USA

## Correction

After publication of the original article [1] an error was noted in the Results section (subheading: Probabilistic model) of this manuscript.

The sentence “A mismatch in segment and breakpoint copy number implies that at least one segment end is left disconnected (Fig. 2 d).”

Should be referring to figure 1d, thus reading:

“A mismatch in segment and breakpoint copy number implies that at least one segment end is left disconnected (Fig. 1 d).”

These errors were introduced during typesetting, thus the publisher apologizes for this error.
