# Supplementary material for: ReMixT: clone-specific genomic structure estimation in cancer
Source: Genome Biol. 2017 Jul 27;18:140. doi: 10.1186/s13059-017-1267-2 (PMC5530528; doi:10.1186/s13059-017-1267-2)
Supplement: Additional file 1 — Supplementary methods, supplementary analyses, and additional experimental results. (PDF 35000 kb) [file 13059_2017_1267_MOESM1_ESM.pdf]

ReMixT: clone-specific genomic structure estimation in cancer

Supplementary Materials

June 26, 2017

Contents

1 The ReMixT Method **2**
1.1 Whole Genome Sequence Data Preprocessing 2
1.1.1 Segmenting the Genome 2
1.1.2 Total Segment Read Counts 2
1.1.3 Haplotype Block Read Counts 3
1.1.4 Allele Specific Segment Read Counts 3
1.2 Statistical Modelling of Segment Read Counts 4
1.2.1 Mixtures of Tumour Genomes Generate Segment Read Counts 4
1.2.2 Raw Major and Minor Copy Number 5
1.2.3 Modelling Total Segment Read Counts 6
1.2.4 Modelling Allele Specific Segment Read Counts 9
1.2.5 GC and Mappability Biases in Total Segment Read Counts 12
1.3 A Probabilistic Model of Genome Structure 15
1.3.1 Total Read Count Likelihood Model 16
1.3.2 Allele Read Count Likelihood Model 16
1.4 A Model of Breakpoint and Segment Copy Number 17
1.5 Structured Variational Inference 19
1.5.1 Segment Copy Number Updates 20
1.5.2 Sum Product and Max Product Algorithms 20
1.5.3 Breakpoint Copy Number Updates 22
1.5.4 Likelihood Variable Updates 22
1.5.5 Parameter Updates 23
1.5.6 Efficient Calculation of Transition Expectations 23
1.5.7 State Space for Segment and Breakpoint Copy Number 24
1.5.8 Assessing Convergence 24
1.6 Identifiability 25
1.7 Initialization 26
1.7.1 Empirical Initialization Strategy for h 26
1.7.2 Initialization of Variational Parameters 27
1.8 Algorithmic Complexity 28

2 Realistic Simulations of Bulk Genome Sequencing **29**
2.1 Simulating Evolutionary Histories 29
2.2 Simulating genome sequence data 31
2.3 Simulation parameters for breakpoint evaluation 32
2.4 Simulation parameters for comparison with existing methods 32
2.5 Post-hoc calculation of breakpoint copy number 32

3 Supplementary Analysis **33**
3.1 Evaluation of Outlier Estimation 33
3.2 Evaluation of Performance for 3 Tumour Clone Mixture 34
3.3 Evaluation of Performance with Increased Sequencing Depth 35
3.4 Comparison of CPU Time and Memory Consumption 36
3.5 Comparison with existing methods for SA501X3F 37

4 Parameters used for existing methods **40**
4.1 THetA2.0 40
4.2 TITAN 40
4.3 CloneHD 40
4.4 Battenberg 40

1 The ReMixT Method

1.1 Whole Genome Sequence Data Preprocessing

The input for ReMixT is a set of concordantly aligned reads, and a set of rearrangement breakpoints predicted from discordant paired end read analysis. Also required is a mapping from each concordantly aligned read to the SNPs contained within that read, together with the allele each read supports for each SNP. Input read alignments and breakpoints are preprocessed to produce a segmentation, total and allele specific read counts for each segment, and tumour specific adjacencies between segments as specified by the set of breakpoints.

1.1.1 Segmenting the Genome

We define a regular segmentation of the genome, and augment this segmentation with breakends of rearrangement breakpoints. Let L_{seg} be the length of regular segments (500 kb for this study). A regular segmentation is created by partitioning each chromosome of length L_{chrom} so as to create segment ends $(0, L_{\text{seg}}, 2L_{\text{seg}}, 3L_{\text{seg}}, ...L_{\text{chrom}})$. Input breakpoints are defined as pairs of breakends, themselves defined as an oriented position in the genome. The set of regular segment ends is augmented by adding all breakends as additional segment ends, to create a final set of N genomic segments to be used by ReMixT (see Figure 1). Let l_n be the length of segment n.

1.1.2 Total Segment Read Counts

Represent each concordant read alignment $r = (r_{\text{start}}, r_{\text{end}}) \in \mathbb{N}^2$ as a pair of positions representing the start and end of the alignment of the read in the genome [1]. Calculate the total segment read count x_n as the number of concordant read alignments fully contained within segment n.

[1] For paired end reads, x is the start of the left end and y the end of the right end.

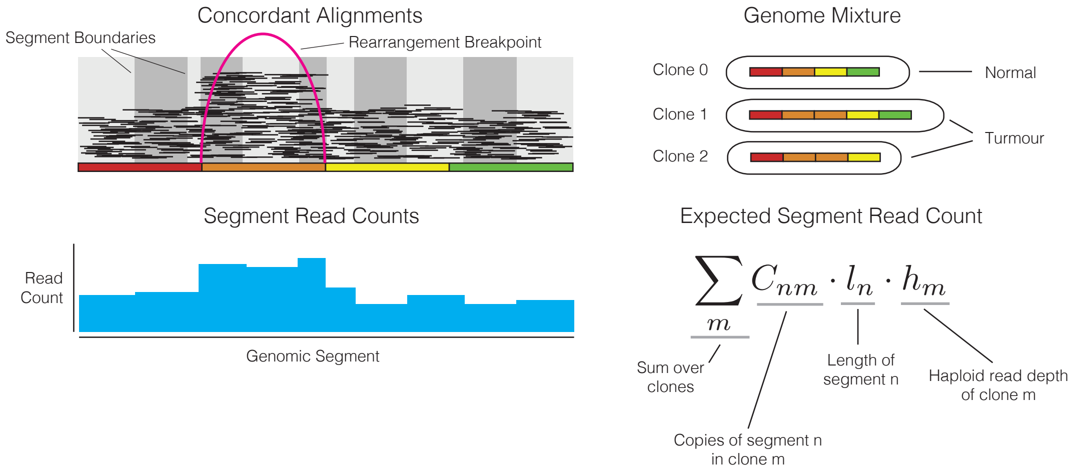

Figure 1: Preprocessing and Modelling Segment Read Counts. Regular segmentation is augmented by breakends of rearrangement breakpoints (alternating grey, top left). Contained concordant reads are counted for each segment (blue histogram, bottom left). Based on a genome mixture (top right), expected segment read count is calculated as the product of segment copy number, segment length, and haploid read depth, summed over all clones.

1.1.3 Haplotype Block Read Counts

Heterozygous germline SNPs can be used to distinguish reads originating from distinct parental alleles. Calculation of allele specific read counts can then be used to estimate allele specific copy number. *Haplotype* information allows for aggregation of read counts from multiple SNPs, increasing statistical strength significantly.

Let $\chi_i \in \{0,1\}$ be a binary indicator representing the two possible alleles of heterozygous SNP i. A *haplotype block* $\eta = (i,k,y), y \in \{0,1\}^k$ is a sequence of alleles $(\chi_i = y_1, .., \chi_{i+k-1} = y_k)$ for k consecutive SNPs starting at i, where the sequence of alleles exist consecutively on the same physical chromosome. The *alternate haplotype block* $\bar{\eta} = (\chi_i = \bar{y}_1, .., \chi_{i+k-1} = \bar{y}_k)$ represents the other of the two parental alleles (here $\bar{\chi} = 1 - \chi$). To infer haplotypes we first predict heterozygous SNPs from a matched normal and then use shapeit2 [4] and a 1000 genomes reference panel.

Calculate haplotype block read counts as follows (see Figure 2). Call a read r as *non-conflicting* with $\eta = (i,k,y)$ if for all $j \in \{i,..,i+k-1\}$ read r matches allele $\chi_{i+j-1} = y_j$. Call a read r as *supporting* of η if it is non-conflicting, and contains at least one SNP j from $j \in \{i,..,i+k-1\}$. Let z_η and $z_{\bar{\eta}}$ be counts of reads that support η and $\bar{\eta}$ respectively.

1.1.4 Allele Specific Segment Read Counts

Let y_{n1} and y_{n2} refer to minor and major allele read counts of segment n respectively, where by convention major and minor are assigned such that $y_{n1} \leq y_{n2}$ [2] [3]. Allele specific segment read counts are calculated from haplotype block read counts by phasing across a segment as follows. For each haplotype block j overlapping segment n, calculate $z_{\eta_{j,n}}$ as the count of reads that a) are

[2]Classification into paternal/maternal is impossible without knowledge of the parental genomes, and somewhat irrelevant in the context of this work.

[3]Note that major/minor does not imply more/less copies in all populations. The major allele may have less copies than the minor allele in a low prevalence subpopulation, even though the combination of populations produces more read counts for the major allele.

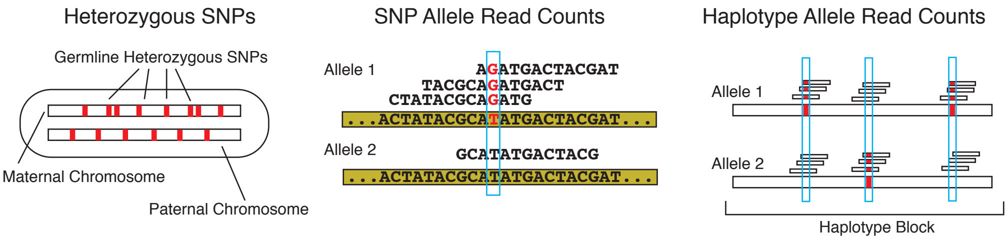

Figure 2: Haplotype Allele Read Counts. Heterozygous SNPs are present on one or the other parental allele in the normal genome (left). SNP allele read counts count reads supporting each allele of a heterozygous SNP (middle). By contrast, haplotype allele read counts are counts of reads supporting any subset of SNP alleles in a haplotype block, for each of the two alleles of that block (right).

contained within segment n, and b) support haplotype η_j. Calculate $z_{\bar{\eta}_{j,n}}$ similarly. Allele specific segment read counts y_{nk} for allele k are calculated as given by Equation 1.

$$\begin{aligned} y_{n1} &= \sum_j \min(z_{\eta_{j,n}}, z_{\bar{\eta}_{j,n}}) \\ y_{n2} &= \sum_j \max(z_{\eta_{j,n}}, z_{\bar{\eta}_{j,n}}) \end{aligned} \quad (1)$$

Note that the total number of allele specific read counts represent a fraction of total reads for a segment, ie $y_{n1} + y_{n2} < x_n$. Also note that we will model the unknown mapping between observed and modeled alleles using a binary indicator variable (see Section 1.3.2).

1.2 Statistical Modelling of Segment Read Counts

In the following section we motivate the likelihood model used by ReMixT. We first describe how we model segment read counts as generated by a mixture of tumour genomes. We then motivate our choice of likelihood model for total and allele specific read count using an analysis of HCC1395 cell line data. Finally, we describe how we account for two well known biases in read count data: GC content and mappability.

1.2.1 Mixtures of Tumour Genomes Generate Segment Read Counts

Let R_m be the number of concordant reads contributed by cell population m in a heterogeneous tumour sample. We assume each of the R_m reads are sampled uniformly from the length L_m of the genome of cell population m. Thus each cell population contributes a specific number of reads per nucleotide, $h_m = \frac{R_m}{L_m}$, to the sequencing experiment. We call h_m the *haploid read depth* of population m since it is the read depth contributed by a single copy of a segment by population m. The haploid read depth encodes both the clonal fractions ρ and the total amount of sequencing. Clonal fractions can be calculated from haploid read depths using Equation 2 [4]. For convenience we use h_m when modelling read counts, and convert back to clonal fractions when necessary.

$$\rho_m = \frac{h_m}{\sum_{m'} h_{m'}} \quad (2)$$

[4]Let N_m be the fractional number of cells sequenced from population m, assuming all nucleotides of those cells are sequenced. Then $R_m = \frac{N_m L_m}{y} = h_m L_m$ where y is the length of sequenced fragments. Thus $N_m = h_m y$, and assuming fragment length y is invariant between cell populations, normalizing h_m is equivalent to normalizing N_m and results in clonal fraction ρ_m.

Let $c_{nm\ell}$ represent the number of copies of allele ℓ of segment n in clone m. The expected total read count contributed by population m for segment n is a linear combination of l_n: the length of the segment, $c_{nm} = \sum_\ell c_{nm\ell}$: the total copy number of the segment in population m, and h_m: the haploid read depth of population m. Thus, the expected total read count μ_n of segment n can be calculated by summing over the M populations in the mixture as given by Equation 3 (see also Figure 1).

$$\mu_n = l_n \sum_m h_m \sum_\ell c_{nm\ell} \tag{3}$$

In practice, real read count data is subject to biases that depend, in part, on the GC content and mappability of the segment. In Section 1.2.5 below, we describe our method for correcting for these biases. We incorporate this correction factor into the segment length, calculating an *effective length* for each segment and using that value in place of the absolute length of the segment.

A similar model of allele specific read count is confounded by our inability to directly and accurately calculate an effective length for allele specific reads. Reads contribute to allele specific counts if they overlap SNPs, and thus counts are dependent on the number of SNPs within a segment, rather than the length of the segment. A single read may overlap one or multiple closely space SNPs, thus read counts cannot be accurately modeled as a proportional to the number of SNPs. For the above reason we take a familiar approach of modelling the expected ratio of major and minor read counts. Our assumption is that major and minor read counts are subject to identical segment specific biases, and thus the expected ratio p_n will depend only on the haploid read depths and allele specific segment copy numbers (Equation 4).

$$p_n = \frac{\sum_m c_{nm1} h_m}{\sum_m \sum_\ell c_{nm\ell} h_m} \tag{4}$$

1.2.2 Raw Major and Minor Copy Number

For visualization purposes, we calculate *raw* major and minor copy number for each segment from observed depths and allele ratios and inferred normal and tumour depth. Let r_n and d_n be the observed allele ratio and depth for segment n. Let $h_{\text{normal}} = h_1$ and $h_{\text{tumour}} = h_2$ be estimated normal and tumour haploid depths respectively. In the situation of multiple tumour clones, h_2 is the sum of haploid depths across all tumour clones. Let $c_{n,1,1}$ and $c_{n,1,2}$ be the known minor and major copy numbers of the normal cells for segment n. Raw minor copy number a_n and raw major copy number b_n can be calculated as given by Equations 5-8.

$$r_n = \frac{y_{n1}}{\sum_k y_{nk}} \tag{5}$$

$$d_n = \frac{x_n}{l_n} \tag{6}$$

$$a_n = \frac{d_n r_n - h_1 c_{n,1,1}}{h_2} \tag{7}$$

$$b_n = \frac{d_n(1 - r_n) - h_1 c_{n,1,2}}{h_2} \tag{8}$$

Plots of raw major and minor copy number across the genome allow for visualization of the dominant integer copy number profile, subclonality as departure from integer copy number, and measurement noise as small scale local variation.

1.2.3 Modelling Total Segment Read Counts

We sought to develop a likelihood model of total read counts with the flexibility to robustly model real tumour sequence data. To that end, we first analysed the HCC1395 cell line using ReMixT, and hand selected a subset of genomic segments with clonally homogeneous copy number (Figure 3). Next, we randomly generated 8 sets of segments with lengths of 10kb, 20kb ... 1280kb, with 10,000 segments generated for each segment length. Segments were required to be fully contained within hand selected regions of the genome with the same allele specific copy number state for the entire length of the segment. We then counted the total and allele specific reads within each segment.

Figure 3: Hand selected clonal segments

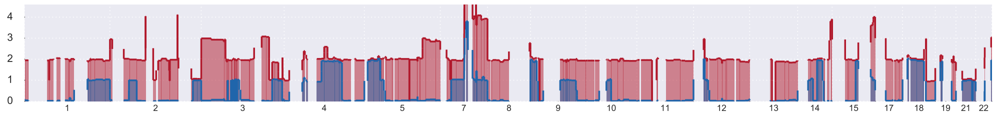

Next we sought to determine the mean variance relationship of total read counts. We fit linear models to log mean read count vs log read count variance, first with segment length fixed (Figure 4 top), then with total copy number fixed (Figure 28 bottom). Additionally, we performed the same analysis for segment read counts corrected using the GC content and mappability biases (read count divided by normalized bias value). In all cases a strong linear relationship in log space indicates a polynomial mean variance relationship. The corrected segment read counts showed consistently lower variance, indicating the bias correction is helping to explain some of the variance in the data. The slope of the best fit lines varied between 1.6 and 1.7, indicating that the corrected data is over-dispersed, and is not Poisson distributed.

Figure 4: Mean variance relationship for segments with fixed segment length (top), and total copy number (bottom). Shown are uncorrected read counts (blue) and read counts with correction for GC and mappability (green).

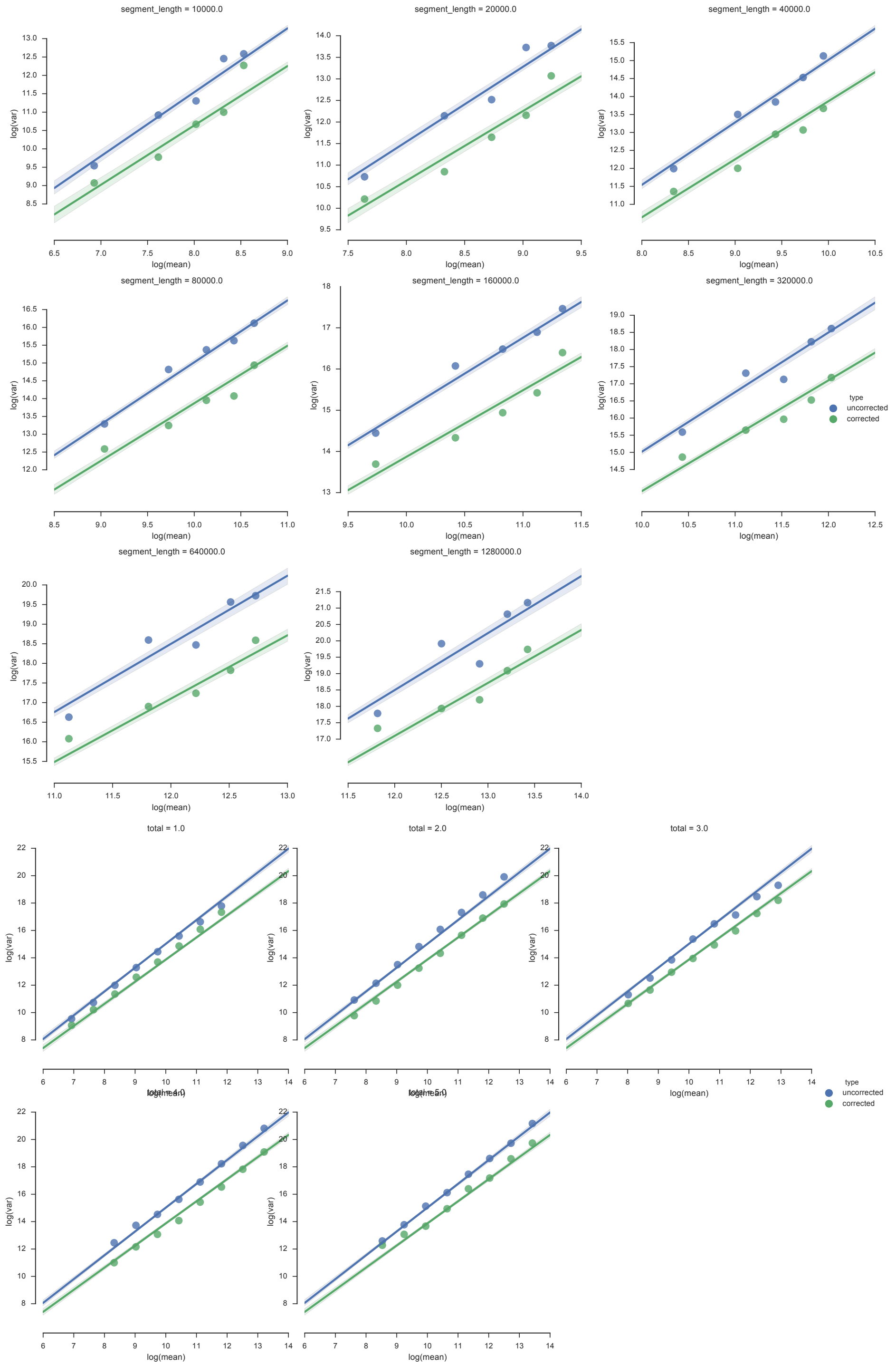

We then experimented with several distributions to determine which distribution would best model the total read count data. As expected, a Poisson distribution showed poor fit as determined by QQ plot (Figure 5). Gaussian and Negative Binomial (equivalently, Gamma-Poisson) distribu-

tions performed slightly better but were unable to model outliers (Figure 6 and 7). A mixture of two Negative Binomial distributions, one with fixed high variance (over-dispersion $r = 10.$), and fixed mixing proportion 0.01, performed significantly better (Figure 8). Based on our analysis, over-dispersion and outliers are both significant aspects of total read count data.

Figure 5: Histogram (top) and QQ plot (bottom) showing fit of total read counts to Poisson distribution.

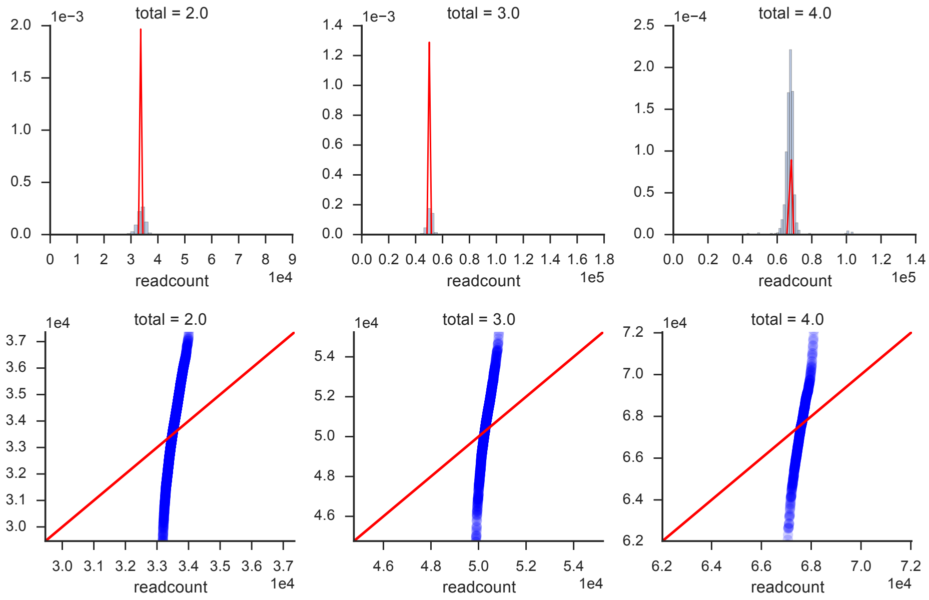

Figure 6: Histogram (top) and QQ plot (bottom) showing fit of total read counts to Gaussian distribution.

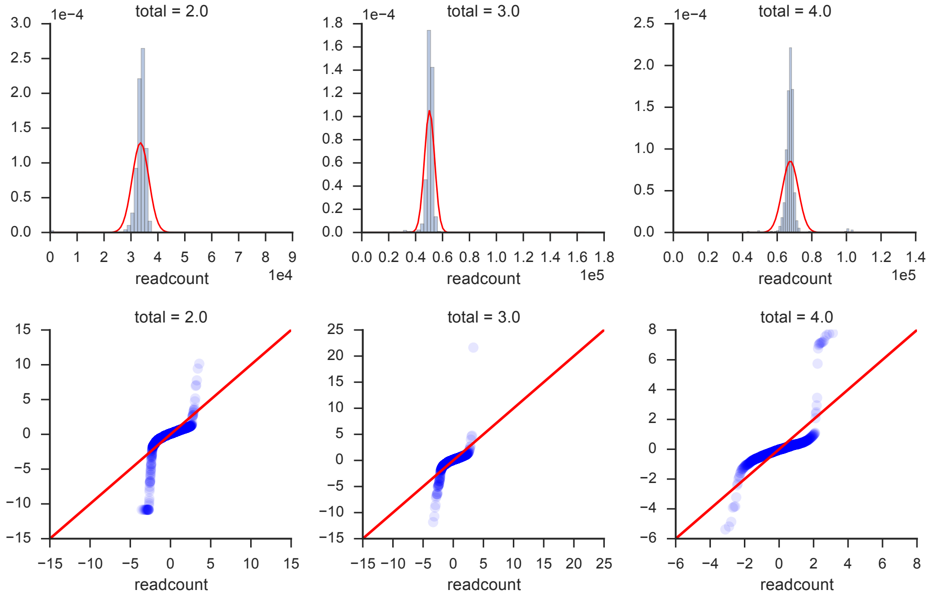

Figure 7: Histogram (top) and QQ plot (bottom) showing fit of total read counts to Negative Binomial distribution.

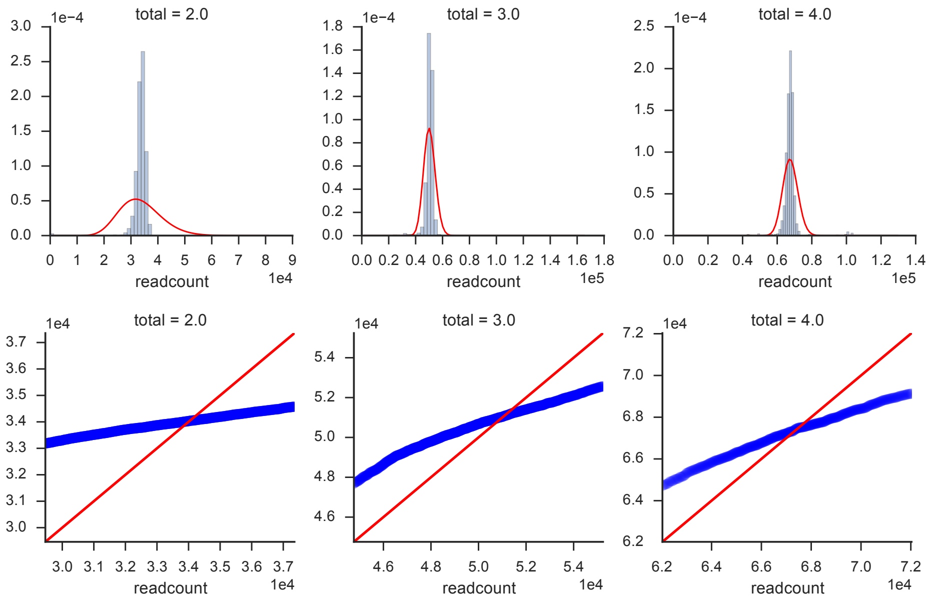

Figure 8: Histogram (top) and QQ plot (bottom) showing fit of total read counts to Negative Binomial mixture distribution.

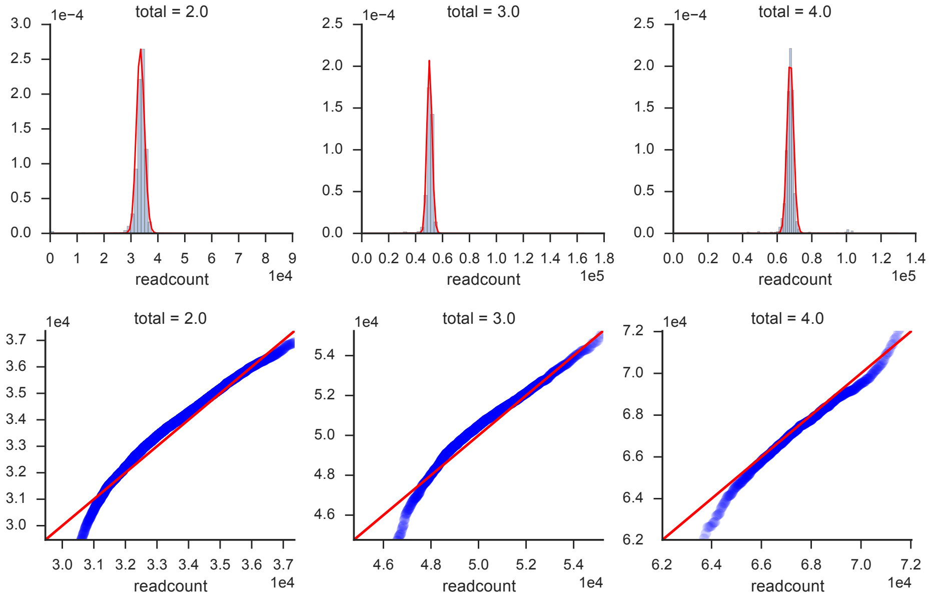

1.2.4 Modelling Allele Specific Segment Read Counts

As with total read counts, we sought to develop a robust likelihood model of allele specific read counts. In general we sought a likelihood model of minor and major allele read counts given an expected ratio of minor and major alleles. Establishing the optimal distribution for allele specific read counts proved less straightforward than for total read counts. An ideal dataset would contain

read counts for which minor + major read counts are fixed at specific values, however such a dataset is difficult to generate from real sequence data as haplotype blocks are of variable length and capture a variable number of reads. Instead, we assumed a Binomial likelihood would best fit the read count data, and sought to determine the extent to which a beta prior would be useful for capturing uncertainty in the true allele ratio generating the data.

We analyzed a subset of the data with segment length of 320kb, major + minor read count of at least 10,000. For these read counts, we expected binomial sampling to comprise a less significant proportion of the variance in the data. Thus we expected the observed minor / major read count ratio would reflect the true allele ratio generating the data. A single Beta distribution was a poor fit for the read count ratio data, as it was unable to robustly model outliers (Figure 9). A mixture of a Beta distribution and a uniform distribution with mixture proportion 0.01 showed a more reasonable fit (Figure 10). As for total read counts, over-dispersion and outliers are both significant aspects of allele read count data.

Figure 9: Histogram (top) and QQ plot (bottom) showing fit of allele ratios to Beta Binomial distribution.

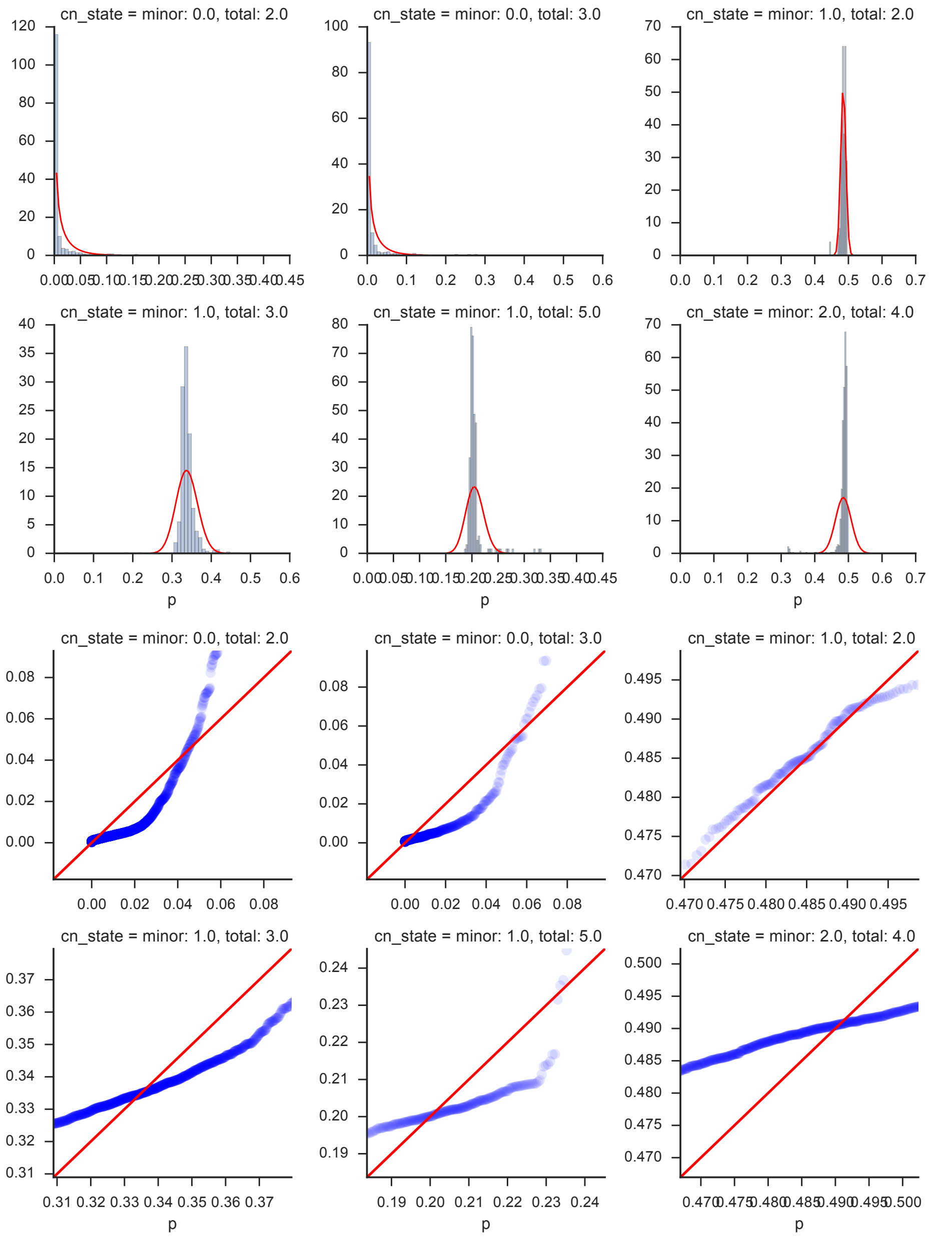

Figure 10: Histogram (top) and QQ plot (bottom) showing fit of allele ratios to Beta Binomial mixture distribution.

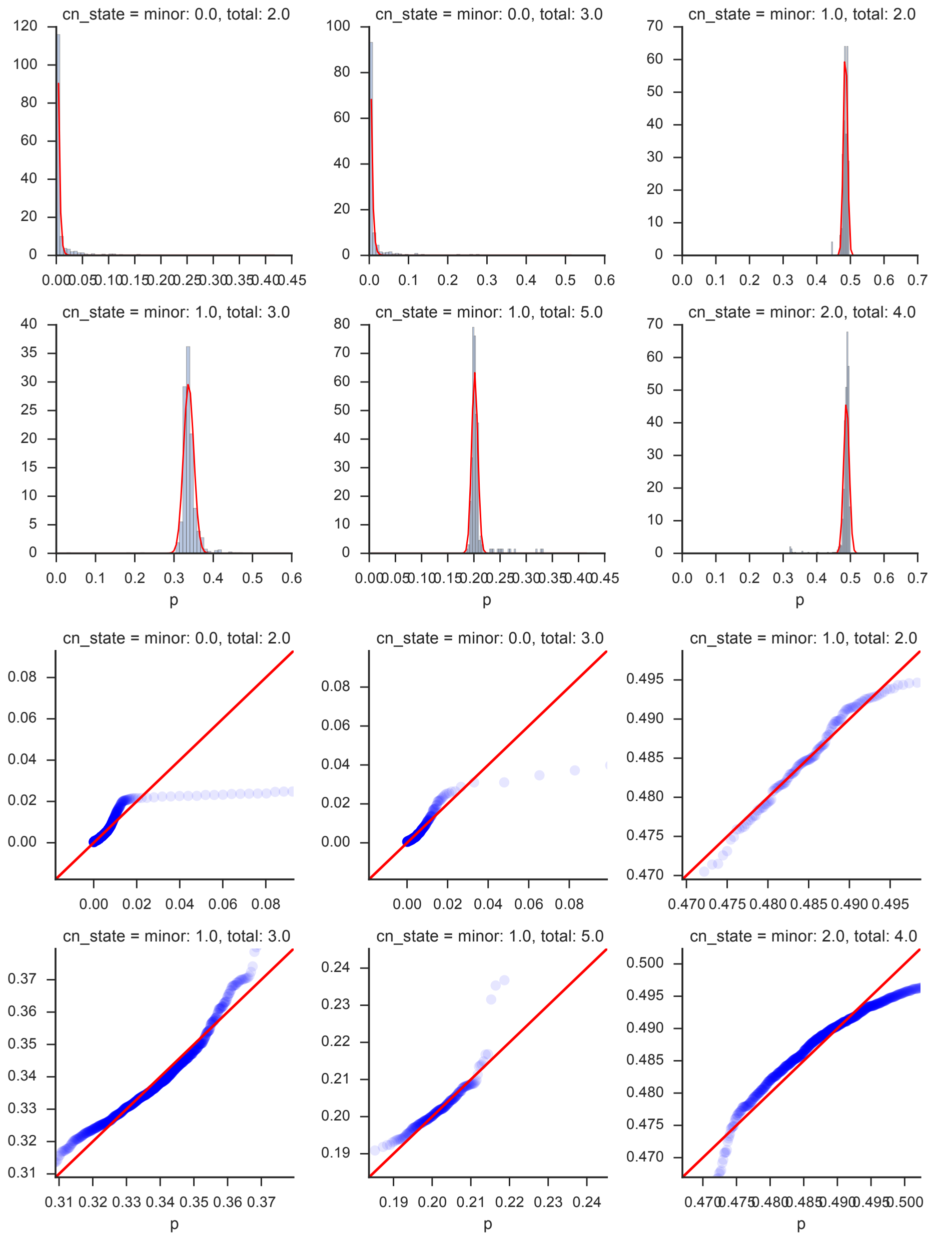

1.2.5 GC and Mappability Biases in Total Segment Read Counts

We use a position specific method of GC and Mappability bias correction as previously described [1]. We provide the following probabilistic interpretation. First, consider an idealized genome with no copy number variation with respect to the reference genome. Let $\delta_{s,l}$ be a categorical random variable associated with the event of observing the read with identity defined by reference start position s and length l. We can write the probability of $\delta_{s,l}$ dependent on biases intrinsic to the read including length l, GC content g, and mappability m. We can think of the probability of observing $\delta_{s,l}$ as dependent on theses three biases, or features (Equation 9). Using the assumptions of the naive bayes classifier, and also assuming each read is equally probable a-priori, the probability of

observing $\delta_{s,l}$ is proportional to the product of the conditionals of each feature (Equation 10). We describe the form and method of calculation of the three conditional probabilities below.

$$p(\delta_{s,l}|l,g,m) = \frac{p(\delta_{s,l})p(l,g,m|\delta_{s,l})}{p(l,g,m)} \tag{9}$$
$$= \frac{p(l|\delta_{s,l})p(g|\delta_{s,l})p(m|\delta_{s,l})}{\sum_{s,l} p(l|\delta_{s,l})p(g|\delta_{s,l})p(m|\delta_{s,l})}$$
$$\propto p(l|\delta_{s,l})p(g|\delta_{s,l})p(m|\delta_{s,l}) \tag{10}$$

Let R be the total number of sequenced reads that fall within the boundaries of any segment. Since $p(\delta_{s,l}|\cdot)$ is assumed small, the expected number of reads $E[x_n] = \mu_n$ contained within segment S_n can be written as given by Equation 11. Let β_n, written as given in Equation 12, be equal to the expected number of reads of a single copy of segment n within a multiplicative constant.

$$\mu_n = R \sum_{s,l \in S_n} p(\delta_{s,l}|l,g,m) \tag{11}$$
$$\propto \sum_{s,l \in S_n} p(l|\delta_{s,l})p(g|\delta_{s,l})p(m|\delta_{s,l}) = \beta_n \tag{12}$$

Now consider the more realistic situation of M clones each with fraction ρ_m and total copy number c_{nm} for clone m, segment n. In this situation, $p(\delta_{s,l})$ is not uniform across the genome, and can be expressed as given by Equation 13.

$$p(\delta_{s,l}) = \frac{\sum_m \rho_m c_{nm}}{L} \qquad \forall (s,l) \in S_n \tag{13}$$
$$L = \sum_n \sum_m \rho_m c_{nm} \tag{14}$$

Thus we can rewrite μ_n as given by Equation 15.

$$\mu_n = \frac{R}{ZL} \beta_n \sum_m \rho_m c_{nm} \tag{15}$$

By comparing Equation 15, with Equation 3, β_n can be interpreted as the bias corrected length of segment n, Z as the total bias corrected length of the reference genome, and $\frac{R\rho_m}{Z}$ as the haploid read depth h_m of clone m.

We now describe the form of each conditional probability and how each is calculated. The distribution of observed fragment lengths $p(l|\delta_{s,l})$ is trivially calculated from the distribution of distances between concordantly aligned paired end reads, and is approximated using a normal distribution.

The distribution of percent GC content of observed reads $p(g|\delta_{s,l})$ can be calculated as follows [1]. Select $10,000,000$ high mappability positions at random within the genome. For each position, calculate the number of reads starting at exactly that position, and the percent GC of an average length fragment starting at that position. Stratify percent GC values into bins. For each bin, calculate the mean fragment rate as the number of fragments starting at a position falling into the given GC bin, divided by the total number of positions for the given GC bin. Finally, create a lowess smoothed distribution of percent GC content of observed reads from a histogram of mean fragment rate per GC bin. See Figure 11 for an example.

Figure 11: Histogram of percent GC content for observed reads (grey bars) and Lowess smoothed density for CR007P (red line)

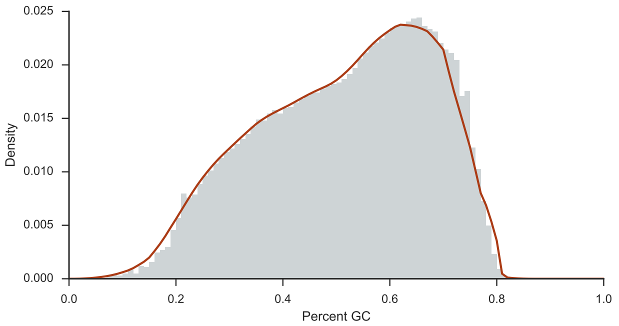

We construct a distribution of read mappability $p(m|\delta_{s,l})$ as follows. We first create a set of pseudo-reads from all k-mers of the reference genome, where k is the length of reads in the sequencing data to be analyzed. Align the set of pseudo-reads back to the reference using the same aligner as will be used for the analysis of the sequencing data. Based on the resulting alignments, classify position s as uniquely mappable or unmappable based on whether the k-mer starting at s was successfully mapped back to position s and only position s. Define the mapping u such that $u(s) = 1$ if s is uniquely mappable otherwise $u(s) = 0$. Finally, define $p(m|\delta_{s,l})$ such that $p(m|\delta_{s,l}) = 1$ if and only if both k length reads for a fragment starting at s with length l are mappable (Equation 16).

$$p(m|\delta_{s,l}) = u(s) \cdot u(s + l - k) \tag{16}$$

We tested our bias correction methods with counts of reads normal blood sample CR007B for randomly generated segments in chromosome 20. We generated 1000 random and potentially overlapping segments of chromosome 20 with segment lengths of 10,000, 100,000, and 1,000,000. We counted reads within these segments, and calculated a bias for each segment based on the GC curve for CR007B. Correcting for GC and Mappability biases as outlined above significantly reduces the variance of read count data for CR007B (Figure 12).

Figure 12: Boxplots of corrected vs uncorrected read depths for 3 segment lengths, 1000 randomly selected segments per length, for normal sample CR007B

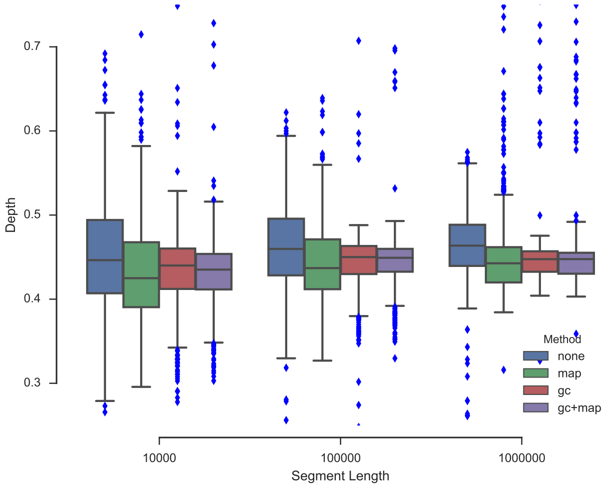

1.3 A Probabilistic Model of Genome Structure

Below we elaborate on the ReMixT model of genome structure described in the main text, including modelling of allele specific read counts and outliers. Figure 13 shows a factor graph representation of the ReMixT model. Variable definitions are shown in Table 1.

Figure 13: Shown is a factor graph of the full ReMixT model. Variables representing segment copy number are organized in a chain from left to right, with c_n, and $c_n + 1$ and adjacent factors shown. Not shown are factors representing priors on u, v, and w, in addition to h and other likelihood parameters θ.

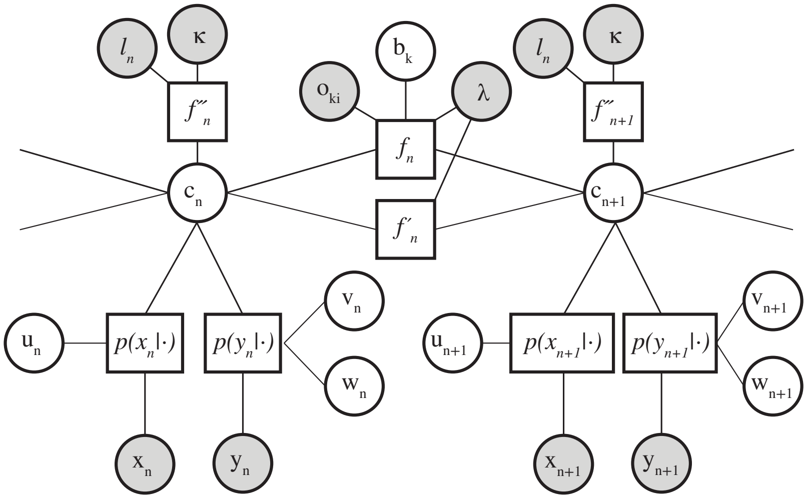

Table 1: Definitions of variables in the ReMixT model

parameter	description
x_n	Observed total read count for segment n
y_n	Observed allele specific read counts for segment n
$c_{nm\ell}$	Copy number of segment n, clone m, allele ℓ
b_k	Breakpoint copy number of breakpoint k
k_n	Index of breakpoint with breakend interposed between segment n and $n+1$
b_{k_n}	Copy number of breakpoint with index k_n
$o_n \in \{-1, +1\}$	Orientation of breakpoint between segment n and $n+1$
$u_n \in \{0, 1\}$	Binary outlier indicator for observed total reads for segment n
$v_n \in \{0, 1\}$	Binary outlier indicator for observed allele specific reads for segment n
$w_n \in \{0, 1\}$	Binary allele swap indicator for segment n
f_n	Total copy number transition factor for segment n to $n+1$
f'_n	Allele specific copy number transition factor for segment n to $n+1$
f''_n	Copy number divergence factor for segment n
λ	Copy number transition penalty parameter
κ	Copy number divergence penalty parameter

1.3.1 Total Read Count Likelihood Model

As motivated above, we use a mixture of two negative binomial distributions to capture both over-dispersion and outliers in total read count data. Let binary variable u_n model whether segment n is an outlier with respect to total read counts. Let $p(x|c, h, l, u, \theta)$ be the likelihood of observed total read counts x given per clone segment copy number c, segment length l, global likelihood parameters θ, per clone haploid read depths h, and outlier status u.

We model the mean of the negative binomials as equal to the expected total read count, as calculated by Equation 3. For homozygously deleted segments in samples with no normal contamination, the expected total read count will be 0, and the negative binomial probability will be undefined. Thus when ReMixT is configured to run on a sample with no normal contamination, we allow the parameters of the negative binomial for the homozygous deletion copy state ($\sum_{m,\ell} c_{m\ell} = 0$) to be a free parameter with positive support, estimated during model fitting.

For convenience, define binary indicators *contam*, set to 1 by the user when normal contamination should be modeled, and *hdel* a binary function that evaluates to 1 when the parameters of the negative binomial should be free to vary for the homozygous deletion state (Equation 17).

$$hdel(c) = \begin{cases} 1 & \text{for } \sum_{m,\ell} c_{m\ell} = 0 \text{ and } contam = 0, \\ 0 & \text{otherwise} \end{cases} \tag{17}$$

Let $\tilde{\mu}(c)$ be the copy number state specific mean parameter of the negative binomial (Equation 18).

$$\tilde{\mu}(c) = \begin{cases} \mu_{hdel} & \text{for } hdel(c) = 1, \\ l \sum_m c_m h_m & \text{otherwise} \end{cases} \tag{18}$$

Parametrize the negative binomial distribution using probability mass function as given by Equation 19.

$$\mathrm{NB}(x|\mu, r) = \frac{\Gamma(r+x)}{\Gamma(x)\Gamma(r)} \left(\frac{r}{r+\mu}\right)^r \left(\frac{\mu}{r+\mu}\right)^x \tag{19}$$

An over-dispersion constant r for the negative binomial is estimated for each combination of *hdel* and u values, for a total of four possible parameters (two when $contam = 1$). Write the likelihood of the total read counts x as given by Equation 20.

$$p(x|c, h, l, u, \theta) = \mathrm{NB}(x|\tilde{\mu}(c), r_{hdel,u}) \tag{20}$$

For the purposes of this study, we use a prior over u_n with $p(u_n = 1) = 0.01$.

Note that CloneHD [7] also uses a negative binomial likelihood for total read counts, though they do not use a mixture for capturing outliers.

1.3.2 Allele Read Count Likelihood Model

As motivated above, we use a mixture of two beta binomial distributions to capture both over-dispersion and outliers in allele read count data. Let binary variables v_n model whether segment n is an outlier with respect to allele specific read counts. Additionally, let binary *allele swap* variable w_n represent whether the observed major and minor allele read counts originate from modeled allele 1 and allele 2 read counts or visa versa. Finally, let $p(y|c, h, v, w, \theta)$ be the likelihood of observed allele specific read counts y given outlier state v, allele swap variable w, and c, θ, and h.

We model the mean of the beta binomials as equal to the expected allele ratio as calculated by Equation 4. For loss of heterozygousity (LOH) segments in samples with no normal contamination, the expected ratio will be 0 or 1, and the beta binomial undefined. Similar to the total read counts, we allow the parameters of the beta binomial to vary freely for the LOH copy number state ($\min_\ell \sum_m c_{m\ell} = 0$) when ReMixT is configured to run on a sample with no normal contamination.

Define binary function loh that evaluates to 1 when the parameters of the beta binomial should be free to vary for the LOH state (Equation 21).

$$loh(c) = \begin{cases} 1 & \text{for } \min_\ell \sum_m c_{m\ell} = 0 \text{ and } contam = 0, \\ 0 & \text{otherwise} \end{cases} \tag{21}$$

Let $\tilde{p}(c)$ be the copy number state specific mean parameter of the negative binomial (Equation 22).

$$\tilde{p}(c) = \begin{cases} p_{loh} & \text{for } loh(c) = 1, \\ \frac{\sum_m c_{m1} h_m}{\sum_m \sum_\ell c_{m\ell} h_m} & \text{otherwise} \end{cases} \tag{22}$$

Parametrize the beta binomial distribution using probability mass function as given by Equation 23.

$$\text{BB}(k|n, p, M) = \frac{\Gamma(n+1)}{\Gamma(k+1)\Gamma(n-k+1)} \frac{\text{Beta}(k + Mp, n - k + M(1-p))}{\text{Beta}(Mp, M(1-p))} \tag{23}$$

Allele specific total reads are defined as $n(y) = y_1 + y_2$ and minor allele reads k depend on allele swap indicator w as given by Equation 24.

$$k(y, w) = \begin{cases} y_1 & \text{for } w = 0, \\ y_2 & \text{for } w = 1. \end{cases} \tag{24}$$

An over-dispersion constant M for the beta binomial is estimated for each combination of loh and v values, for a total of four possible parameters (two when $contam = 1$). Write the likelihood of the total read counts x as given by Equation 25.

$$p(y|c, h, v, w, \theta) = \text{BB}(k(y, w)|n(y), \tilde{p}(c), M_{loh,v}) \tag{25}$$

For the purposes of this study, we use a prior over v_n with $p(v_n = 1) = 0.01$. We use a uniform prior for w_n.

Note that CloneHD [7] also uses a beta binomial likelihood for allele specific read counts, though they do not use a mixture for capturing outliers. OncoSNP-SEQ [12] uses a binomial uniform mixture distribution to capture outliers. Our selected allele specific likelihood model can thus be seen as a combination of concepts from both CloneHD and OncoSNP-SEQ.

1.4 A Model of Breakpoint and Segment Copy Number

Let $f(c, c', b|o, \lambda)$ be the a transition factor for copy numbers c and c' of adjacent segments, and copy number b of a breakpoint with breakend interposed between those segments with orientation o. As a mathematical convenience, we represent the orientation of a breakend interposed between segment n and $n+1$ with value $o = -1$ if the breakend is incident to the end of segment n, and $o = +1$ if the breakend is incident to the start of segment $n + 1$. For simplicity we first describe f in the context of a single clone model. Without a breakpoint, it is reasonable to assume that f is a function

of the absolute difference in copy number between adjacent segments $|c - c'|$. Dependant on its orientation, positive copy number for a breakpoint may explain some or all of the difference in copy number between adjacent segments. Thus we model f as a function of $t(c, c', b, o) = |c - c' - o \cdot b|$, where orientation $o \in \{-1, +1\}$ as described above.

We can think of t as measuring the number of *telomeres*, or free segment ends unconnected to another segment end in the model. Our objective is to minimize the number of telomeres as part of our optimization, and we can think of $\log f$ as a penalty on the use of telomeres by the model. A quadratic penalty ($\log f \propto t^2 + \text{const}$) may encourage the model to fit a series of smaller transitions, where a single large transition is more appropriate. A constant penalty for any difference ($\log f \propto \delta(t > 0) + \text{const}$) may encourage a single transition when in fact a series of transitions is true. Thus we choose a linear penalty in the absolute value of the copy number difference. The specific form of f used by ReMixT is given by Equation 26, where λ is a constant governing the strength of the penalty.

$$\log f(c, c', b|o, \lambda) \quad = \quad \lambda \cdot t(c, c', b, o) + \text{const} \tag{26}$$

Below we extend the above transition model to the more general case of multiple clones and allele specific copy number. In modelling multiple clones, ReMixT makes an implicit phylogenetic assumption that the two clones diverged immediately before the first genomic change. Although this assumption may not be true for some tumours, the resulting formulation proves to be computationally tractable. Specifically, we calculate the penalty on the number of telomeres as the sum of those telomeres across clones (Equation 27).

$$\log f(c, c', b|o, \lambda) \quad = \quad \lambda \sum_m t(c_m, c'_m, b_m, o) \tag{27}$$

The formula for f given by Equation 27 calculates a penalty based on total copy number. However, it is possible for total copy number to be constant, and allele specific copy number to oscillate in order to overfit to allele read counts. We thus introduce an additional factor f' in our transition model based solely on allele specific copy number, and excluding breakpoints (Equation 28). We can think of f' as calculating the minimum allele specific copy number transition out of the two possible phasings of the adjacent alleles. From this we subtract the total copy number transition, without accounting for breakpoints.

$$\begin{aligned}\log f'(c, c'|\lambda) \quad &= \quad \lambda \min\left\{\sum_{m,\ell} \left|c_{m\ell} - c'_{m\ell}\right|, \sum_{m,\ell} \left|c_{m\ell} - c'_{m\bar{\ell}}\right|\right\} \\ &\quad -\lambda \left|\sum_{m,\ell} c_{m,\ell} - \sum_{m,\ell} c'_{m,\ell}\right| \end{aligned} \tag{28}$$

Note that our choice of penalty f' is motivated by experience with an initial version of the model in which allele specific copy number, allele swap variables, and breakpoint copy number are tightly coupled, and for which inference was less tractable and initialization difficult.

Finally, we define a factor $f''(c|l, \kappa)$ over copy number states to represent the belief that subclonal segments should be relatively rare. Clone specific copy number adds significant flexibility to the model, resulting in over-fitting to the likelihood, a problem that is addressed using a factor that penalizes subclonality. The form of the penalty we use is segmentation invariant, and amounts to a length scaled linear penalty on the log likelihood, with scaling factor κ (Equation 29)

$$\log f''(c|l, \kappa) \quad = \quad \kappa l \left(\sum_\ell \max_m c_{m\ell} - \min_m c_{m\ell}\right) \tag{29}$$

Although it would be preferable to place a prior over the proportion of the entire genome that is subclonal, such a prior would result in a less tractable model. In practice, we try several settings of κ (each of $1e^{-6}$, $1e^{-7}$, and $1e^{-8}$ for this study), and select the solution that both maximizes the posterior and satisfies a threshold on the proportion of divergent segments (50% for this study).

Let k_n be the index of a breakpoint with breakend at the end of segment n or the start of segment $n+1$. For ease of presentation, k_n for adjacent segments with no incident breakend between them will refer to a null breakpoint with copy number fixed at 0. Furthermore, b_k will refer to the copy number of breakpoint k, while b_{k_n} will refer to the copy number of the breakpoint incident at the boundary between segment n and $n+1$, with similar indexing for orientation o. Write the joint probability of the observed read counts and segment and breakpoint copy number as given by Equation 30.

$$\begin{aligned} p(X, C, B, U, V, W | h, L, \theta) &= \frac{1}{Z} \prod_{n=1}^{N} p(x_n | c_n, h, l_n, u_n, \theta) \times p(u_n) \\ & \prod_{n=1}^{N} p(y_n | c_n, h, v_n, w_n, \theta) \times p(v_n) \\ & \prod_{n=1}^{N-1} f(c_n, c_{n+1}, b_{k_n} | o_n, \lambda) \times \\ & \prod_{n=1}^{N-1} f'(c_n, c_{n+1} | \lambda) \times \\ & \prod_{n=1}^{N} f''(c_n | l_n, \kappa) \end{aligned} \tag{30}$$

1.5 Structured Variational Inference

In contrast to HMMs, exact inference in the ReMixT model is intractable due to the additional dependencies resulting from modelling the long range connectivity of breakpoints. We thus turn to Variational Inference as a method for performing approximate inference for the ReMixT model. A general discussion of Variational Inference is presented in the main text. In the following section we show how Variational Inference is applied to the full ReMixT model described above.

We approximate the posterior $p(C, B, U, V, W, h, \theta | X, L)$ using a distribution q with factorization given by Equation 31.

$$q(C, B, U, V, W, h, \theta) = q(h) q(\theta) q(C) \prod_{k} q_k(b_k) \prod_{n} q_n(u_n) \prod_{n} q_n(v_n) \prod_{n} q_n(w_n) \tag{31}$$

Taking a variational Expectation Maximization (EM) approach, we specify the distributional form of $q(h)$ and $q(\theta)$ to be the dirac delta function, and compute point estimates for those parameters. The distributional forms of the remaining factors arise naturally from the application of the variational updates (Equation 32). In general, the factors of the discrete variables are categorical, with the exception of $q(C)$ which has an HMM topology as described in the main text.

$$\log q^*(z_j) = \mathbb{E}_{\prod_{j \neq i} q_j(z_j)} [\log p(x, z)] + \text{const} \tag{32}$$

1.5.1 Segment Copy Number Updates

Assuming uniform priors over h and θ, we can write the posterior as given by Equation 33.

$$\log p(X, C, B, U, V, W, h, \theta | L) = \log p(X, C, B, U, V, W | h, \theta, L) + \text{const} \tag{33}$$

Updates to $q(C)$ are calculated as given by Equation 34.

$$\begin{aligned}
\log q^*(C) &= \sum_{B,U,V,W} \int_{h,\theta} q(B, U, V, W, h, \theta) \log p(X, C, B, U, V, W | h, \theta, L) + \text{const} \\
&= \sum_n \zeta_n(c_n) + \sum_{n=1}^{N-1} \zeta_n(c_n, c_{n+1}) + \text{const} \qquad (34) \\
\zeta_n(c_n) &= \log f''(c_n | l_n, \kappa) + \\
&\quad \sum_u q_n(u) \log p(x | c, h, l, u, \theta) + \\
&\quad \sum_v \sum_w q_n(v) q_n(w) \log p(y | c, h, v, w, \theta) \qquad (35) \\
\zeta_n(c_n, c_{n+1}) &= \sum_b q_n(b) \log f(c_n, c_{n+1}, b | o_n, \lambda) + \\
&\quad \log f'(c_n, c_{n+1} | \lambda) \qquad (36)
\end{aligned}$$

Note that $q_n(b)$ refers to the variational distribution over breakpoint copy number for the breakpoint incident between segment n and $n+1$. If no such breakpoint exists, $q_n(b = 0) = 1$ and $q_n(b > 0) = 0$. By inspection, the probability distribution $q^*(C)$ given by Equation 34 has a chain topology equivalent to an HMM. The emission of the HMM is a function of the log divergence factor and the expectation of the read count log likelihood taken over likelihood related variables u, v, and w. The transition of the HMM is a combination of the allele transition and the expectation of the breakpoint related transition over possible breakpoint copy numbers (Equations 35 and 36). The sum product algorithm (Section 1.5.2) can be used to calculate the single and pairwise posterior marginal probabilities of $q(C)$. Single and pairwise posterior marginals will be useful for efficient updates of other factors of the variational approximation as shown below. We denote the posterior marginal probability that $c_n = c$ by $\gamma_n(c)$, and the pairwise posterior marginal probability that $c_n = c$ and $c_{n+1} = c'$ by $\gamma_n(c, c')$.

1.5.2 Sum Product and Max Product Algorithms

Consider a probability model of a set of N variables c_n with chain structure that can be factorized as given by Equation 37.

$$p(\mathbf{c}) = \frac{1}{Z} \prod_n a(c_n) \prod_{n=1}^{N-1} b(c_n, c_{n+1}) \tag{37}$$

The posterior marginal probability $\gamma_n(c)$ is the probability that variable n is in state c marginalizing over the states of all other variables (Equation 38). Similarly the pairwise posterior marginal probability $\gamma_n(c, c')$ is the probability variable n and $n+1$ are in state c and c' respectively,

marginalizing over the states of all other variables (Equation 39).

$$\gamma_n(c) = \sum_{\mathbf{c}\backslash\{c_n\}} p(\mathbf{c}) \tag{38}$$

$$\gamma_n(c, c') = \sum_{\mathbf{c}\backslash\{c_n, c_{n+1}\}} p(\mathbf{c}) \tag{39}$$

Efficient computation of both marginal probabilities is possible using the sum product algorithm [2]. In brief, we compute *messages* $\alpha(c_n)$ and $\beta(c_n)$ in the forward and backward directions on the chain motivated by a variable elimination strategy for reordering the summation over individual variables from Equations 38 and 39 [2]. Messages can be calculated recursively as given by Equations 40 and 41 respectively.

$$\begin{aligned}
\alpha(c_1) &= a(c_1) \\
\alpha(c_n) &= a(c_n) \sum_{c_{n-1}} b(c_{n-1}, c_n) \left[a(c_{n-1}) \sum_{c_{n-2}} ... \right] \\
&= a(c_n) \sum_{c_{n-1}} b(c_{n-1}, c_n) \alpha(c_{n-1})
\end{aligned} \tag{40}$$

$$\begin{aligned}
\beta(c_N) &= 1 \\
\beta(c_n) &= \sum_{c_{n+1}} b(c_n, c_{n+1}) a(c_{n+1}) \left[\sum_{c_{n+2}} ... \right] \\
&= \sum_{c_{n+1}} b(c_n, c_{n+1}) a(c_{n+1}) \beta(c_{n+1})
\end{aligned} \tag{41}$$

Importantly, we require the normalization constant Z, which can be calculated directly from the forward messages as given by Equation 42.

$$Z = \sum_{c_N} \alpha(c_N) \tag{42}$$

Finally, we can calculate the posterior marginals and pairwise posterior marginals as given by Equations 43 and 44.

$$\gamma_n(c) = \frac{1}{Z} \alpha(c_n) \beta(c_n) \tag{43}$$

$$\gamma_n(c, c') = \frac{1}{Z} \alpha(c_n) b(c_n, c_{n+1}) a(c_{n+1}) \beta(c_{n+1}) \tag{44}$$

The max product algorithm seeks the most probably sequence of states $\mathbf{c}^{\max}$ for the N variables in $\mathbf{c}$ [2]. Calculation of the most probable sequence can be motivated by replacing sum with maximum in the definition of the forward messages given by Equation 40, as given by Equation 45.

$$\begin{aligned}
\omega(c_1) &= a(c_1) \\
\omega(c_n) &= a(c_n) \max_{c_{n-1}} \left[b(c_{n-1}, c_n) \left[a(c_{n-1}) \max_{c_{n-2}} ... \right] \right] \\
&= a(c_n) \max_{c_{n-1}} \left[b(c_{n-1}, c_n) \omega(c_{n-1}) \right]
\end{aligned} \tag{45}$$

After recursively calculating ω for each variable, we then calculate the most likely state for the last variable in the sequence, variable N, as given by Equation 46. We then backtrack to find the sequence of states that resulted in $c_N^{\max}$ as given by Equation 47.

$$c_N^{\max} = \operatorname*{argmax}_{c_N} \omega(c_N) \tag{46}$$

$$\begin{aligned} c_n^{\max} &= \operatorname*{argmax}_{c_n} \left[b(c_n, c_{n+1}^{\max}) \left[a(c_n) \max_{c_{n-1}} ... \right] \right] \\ &= \operatorname*{argmax}_{c_n} \left[b(c_n, c_{n+1}^{\max}) \omega(c_n) \right] \end{aligned} \tag{47}$$

In a practical implementation, all probabilities are stored as log probabilities to avoid underflow errors, and summations of probabilities use the well known log sum exponential trick.

1.5.3 Breakpoint Copy Number Updates

Applying Equation 32 to $q_k(b_k)$ results in Equation 48.

$$\begin{aligned} \log q_k^*(b_k) &= \sum_C q(C) \log p(X, C, B, U, V, W | h, \theta, L) + \text{const} \\ &= \sum_{n:k_n=k} \sum_c \sum_{c'} \gamma_n(c, c') \log f(c, c', b_k | o_n, \lambda) + \text{const} \end{aligned} \tag{48}$$

Efficient calculation of the expectation is facilitated by the fact that the relevant factor of the posterior depends only the copy number of incident segments situated to either side of each breakend. The expectation is a summation over all other settings of the non-incident segment copy number variables, akin to marginalization in an HMM. Thus we can efficiently calculate the pairwise posterior marginals ahead of time and use them in place of full summation over all other HMM variables in the calculation of each $\log q_k^*(b_k)$.

1.5.4 Likelihood Variable Updates

Updates for $q_n(u_n)$, $q_n(v_n)$, and $q_n(w_n)$ are given by Equations 49, 50, and 51.

$$\log q_n^*(u_n) = \sum_c \gamma_n(c) \log p(x_n | c, h, l, u_n, \theta) + \log p(u_n) + \text{const} \tag{49}$$

$$\log q_n^*(v_n) = \sum_c \sum_w \gamma_n(c) q_n(w) \log p(y_n | c, h, v_n, w, \theta) + \log p(v_n) + \text{const} \tag{50}$$

$$\log q^*(w_n) = \sum_c \sum_v \gamma_n(c) q_n(v) \log p(y_n | c, h, v, w_n, \theta) + \text{const} \tag{51}$$

As for updates to $q_k(b_k)$, we have replaced a expectation over all individual variables of C with the posterior marginal $\gamma_n(c)$ of the relevant variable c_n.

1.5.5 Parameter Updates

As stated in the main text, since the entropy of a delta function is constant, optimal estimates of h and θ involve minimizing only the $\mathbb{E}_q[\log p(x,z)]$ *variational energy* term of the ELBO.

$$
\begin{aligned}
\mathbb{E}_q[\log p(x,z)] &= \sum_{C,U,V,W} q(C,U,V,W,h,\theta)\log p(X,C,B,U,V,W|h,\theta,L) \\
&= \sum_n \sum_c \sum_u \gamma_n(c) q_n(u) \log p(x_n|c,h,l,u,\theta) \\
&= \sum_n \sum_c \sum_v \sum_w \gamma_n(c) q_n(v) q_n(w) \log p(y_n|c,h,v,w,\theta)
\end{aligned}
$$

Parameters to be updated include h, $r_{hdel,u}$, $M_{loh,v}$, μ_{hdel}, and p_{loh}. To update h, we compute derivatives and use the quasi-newton L-BFGS-B method [3] to find a local minima (`scipy.optimize.fmin_l_bfgs_b` function in python). We use stochastic optimization to facilitate faster updates, optimizing the variational energy for a mini-batch of 200 segments selected uniformly and at random from the full set of segments. For the remaining parameters we use brute force grid search followed by the Nelder-Mead simplex algorithm to *polish* the result (the `scipy.optimize.brute` function in python). Stochastic optimization is also used for the additional likelihood parameters. However, since the additional parameters are outlier and copy number state specific, selecting mini-batches uniformly and at random will perform poorly. Thus for these parameters, mini-batches of segments are selected at random with probability proportional to the posterior marginal probabilities over the relevant outlier and copy number states, as calculated from the current variational approximation. Following calculation of an updated parameter, the update is checked using full calculation of the variational energy and rejected if it would result in increase of the ELBO.

1.5.6 Efficient Calculation of Transition Expectations

The complexity of the described variational method is partially dependent on the form of the transition matrix / function f. For Equation 34, we are required to efficiently compute an expectation with respect to $q(b_k)$, a variational distribution over breakpoint copy number b_k. Symmetry allows for efficient computation with $\mathcal{O}(c_{\max}^2)$ operations (Equation 52).

$$
\begin{aligned}
f(c,c') &= \sum_b q(b)|c-c'-ob| \\
&= \sum_b q(b)|d-ob| = f(d)
\end{aligned} \tag{52}
$$

For Equation 48, we can use a similar trick for efficient computation (Equation 53).

$$
\begin{aligned}
f(b) &= \sum_c \sum_{c'} \gamma(c,c')|c-c'-ob| \\
&= \sum_d |d-ob| \sum_{\substack{c,c' \\ c-c'=d}} \gamma(c,c')
\end{aligned} \tag{53}
$$

1.5.7 State Space for Segment and Breakpoint Copy Number

We model a combined state space of segment copy number for all clones and both alleles as given by Equation 54. The state space is restricted to allow a maximum of 1 copy number difference between clones for each allele. A further restriction prevents redundant pairs of states for which one state can be obtained by swapping the alleles of another state.

$$\begin{aligned}
\mathcal{S} &= \{0...c_{\max}\} \\
\mathcal{T} &= \{(c_0, c_1) \in \mathcal{S} \times \mathcal{S}, c_0 + c_1 \leq c_{\max}, c_0 \leq c_1\} \\
\mathcal{U} &= \left\{c \in \mathcal{T}^M, c \in \mathcal{X}\right\} \\
\mathcal{X} &:= \forall \ell : \max_m c_{m\ell} - \min_m c_{m\ell} \leq 1 \,\text{and} \\
& \quad\ \exists m : c_{m2} < c_{m1} \,\text{or}\, \forall m : c_{m2} \leq c_{m1}
\end{aligned} \tag{54}$$

The state space for breakpoint copy number is defined similarly, without distinct copy numbers per allele (Equation 55).

$$\begin{aligned}
\mathcal{S} &= \{0...c_{\max}\} \\
\mathcal{U} &= \left\{c \in \mathcal{S}^M, c \in \mathcal{X}\right\} \\
\mathcal{X} &:= \forall \ell : \max_m c_{m\ell} - \min_m c_{m\ell} \leq 1 \,\text{and} \\
& \quad\ \exists m : c_{m2} < c_{m1} \,\text{or}\, \forall m : c_{m2} \leq c_{m1}
\end{aligned} \tag{55}$$

1.5.8 Assessing Convergence

We calculate the ELBO at each update to assess convergence (Equation 56). In practice we perform 5 overall updates. Each overall update first updates the discrete variables with order W, C, B, U, V for 5 iterations, after which each parameter is updated once. After these updates, the difference in ELBO of the last overall update is retained for assessment of convergence and evaluation of the quality of the solution.

$$\text{ELBO} = \sum q \log p - \sum q \log q \tag{56}$$

$$\begin{aligned}
\sum q \log p &= \sum_n \sum_c \sum_u \gamma_n(c) q_n(u) \log p(x_n | c, u, \theta) \\
&+ \sum_n \sum_c \sum_v \sum_w \gamma_n(c) q_n(v) q_n(w) \log p(y_n | c, v, w, \theta) \\
&+ \sum_n \sum_u q_n(u) \log p(u) + \sum_n \sum_v q_n(v) \log p(v) \\
&+ \sum_{n=1}^{N-1} \sum_c \sum_{c'} \sum_b \gamma_n(c, c') q_n(b) \log f(c, c', b | o_n, \lambda) \\
&+ \sum_{n=1}^{N-1} \sum_c \sum_{c'} \gamma_n(c, c') \log f'(c, c' | \lambda) + \\
&+ \sum_n \sum_c \gamma_n(c) \log f''(c | l_n, \kappa)
\end{aligned} \tag{57}$$

$$
\begin{aligned}
\sum q \log q \quad = \quad & \sum_n \sum_c \gamma_n(c) \log \zeta_n(c) + \\
& + \sum_n \sum_c \sum_{c'} \gamma_n(c, c') \log \zeta_n(c, c') + \\
& + \sum_k \sum_b q_k(b) \log q_k(b) \\
& + \sum_n \sum_u q_n(u) \log q_n(u) \\
& + \sum_n \sum_v q_n(v) \log q_n(v) \\
& + \sum_n \sum_w q_n(w) \log q_n(w)
\end{aligned} \tag{58}
$$

1.6 Identifiability

By inspection of the Equations 18 and 22, the ReMixT likelihood suffers from poor identifiability. Haploid read depths h may be halved and copy numbers doubled with no effect on the likelihood (Figure 14). Identifiability issues are not specific to ReMixT, and are a known problem for any method attempting copy number inference from sequencing data [10, 8, 11]. High confidence estimates of ploidy would require strong prior assumptions or additional experiments measuring the sizes of nuclei.

Figure 14: Show is the ELBO (y-axis, top) and average ploidy (y-axis, bottom) for values of h_{tumour} (x-axis). Verticle lines show local maximum of the ELBO with respect to h_{tumour}.

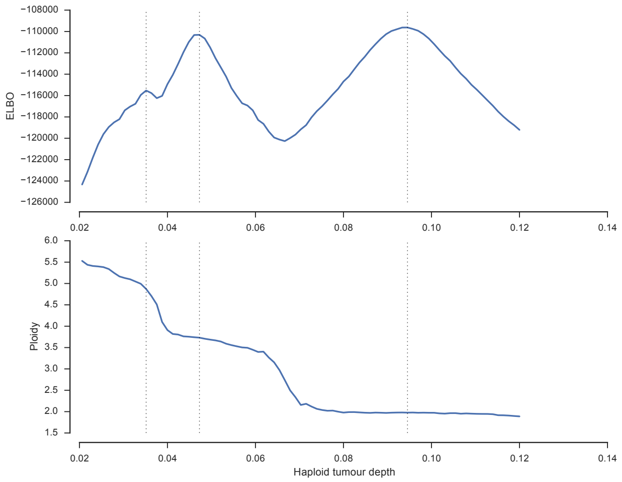

In practice, analysis with ReMixT usually involves per sample manual ploidy curation. To facilitate quick assessment of ploidy values, ReMixT produces a series of plots (Figure 15) for each initialization of h (Section 1.7.1). The plots are designed to allow for quick assessment of the amount of evidence for the ploidy value associated with each initialization of h, compared to a solution with half the ploidy and double the haploid tumour read depth, h_{tumour}. For instance, in the ploidy plot shown in Figure 15, integer copy number calls fall on the modes of the major and minor read depth distributions. However, almost no segments coincide with read depths that would represent copy number 1 or 3, indicating that there is little evidence for the solution shown over the solution with half the ploidy.

Figure 15: Ploidy analysis plot. The centre scatter plot shows segment major (x-axis) and minor (y-axis) read depth colored by chromosome. At right and above the scatter plot are the densities of minor and major read depths respectively. Dashed lines on the scatter plot and density plots annotate read depth values associated with integer copy number calls for this solution, starting at zero copies. The bottom genome plot shows raw major (red) and minor (blue) copy number, with a density plot to the right of the genome plot. In the top right are the value h_{normal}, h_{tumour}, and ploidy.

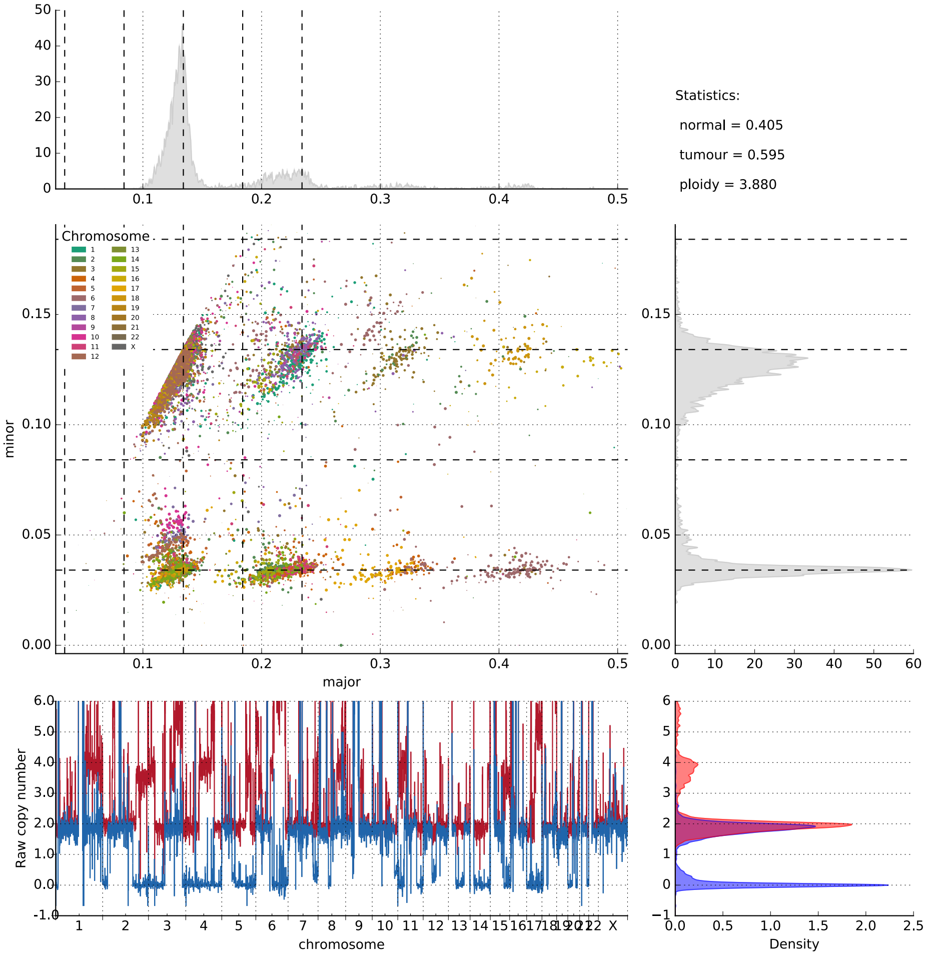

In addition to identifiability issues with overall tumour ploidy as discussed above, identifiability issues also exist for clone specific ploidy. We approach the clone specific ploidy problem by incorporating our prior belief that clone specific copy number changes are rare into our probability model of clone specific genome structure (Equation 29).

1.7 Initialization

1.7.1 Empirical Initialization Strategy for h

Variational inference methods are prone to getting stuck in local optima. ReMixT relies on an empirical strategy to initialize the h parameter to values that represent reasonable solutions within the context of cancer genomics. Our strategy derives from an analysis of the distribution of minor read depths. Let r_n and d_n be the observed allele ratio and depth for segment n calculated as given

in Equations 5 and 6. The minor read depth can be calculated as given by Equation 59.

$$m_n = d_n r_n \tag{59}$$

We assume that a significant proportion (>5%) of the tumour genome is characterized by ancestral loss of heterozygousity (LOH). In other words, a significant number of segments will have 0 minor copies ubiquitously throughout the tumour population. The minor read depth of these segments originates exclusively from the minor allele of the normal contaminating cells. Given these assumptions, the first mode of the minor read depth distribution (solid green line in Figure 16) originates from segments with 0 minor copies across all cells, and the location of that mode corresponds to the haploid read depth of the normal cells.

Furthermore, if a significant proportion of the tumour genome has 1 copies of the minor allele, then those segments will form an additional mode of the minor read depth distribution. For many tumours, especially those with low heterogeneity and low noise, the second highest mode in the read depth distribution originates from those 1 minor copy segments. For these simpler tumour datasets, the read depth at the second highest mode is equal to the normal haploid read depth plus the tumour haploid read depth. However, for more complex tumours with heterogeneity or a recent genome doubling, identifying the 1 minor copy mode may be more difficult.

To initialize h in the ReMixT model, we first identify modes of the read depth distribution using k-means with k=5 applied to segment read depths resampled by weights proportional to the segment's length. We then calculate the first minor mode of the read depth distribution and use that as the haploid normal read depth h_{normal}. Next we enumerate the additional modes of the read depth distribution as candidates for the haploid tumour depth h_{tumour}. To account for a possible recent genome doubling, half the value of each potential haploid tumour depth are also added to the pool of candidates. Finally, we calculate initial $h = (h_{\text{normal}}, \rho \cdot h_{\text{tumour}}, (1-\rho) \cdot h_{\text{tumour}})$ where $\rho \in \{0.45, 0.3, 0.2, 0.1\}$.

Figure 16: Distribution of segment read depths for patient 3, ROv2. Shown is total read depth (gray), minor read depth (blue), and major read depth (red). A solid green line annotates the mode of the minor read depth distribution putatively corresponding to segments with 0 minor copies. Dashed green lines annotate modes possibly corresponding to segments with 1 minor copies.

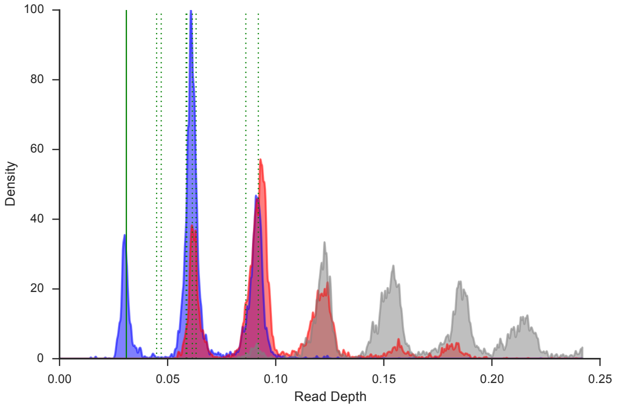

1.7.2 Initialization of Variational Parameters

The variational parameters are initialized as follows. For $q(B)$, all breakpoint states for which breakpoint copy number is > 1 in any clone are given 0 probability with the remaining probability mass distributed uniformly across the remaining states. The posterior marginals of $q(C)$ are

initialized to be uniform across all states. The allele swap probabilities $q(w_n)$ are initialized to be uniform across both states. Both sets of outlier probabilities $q(u_n)$ and $q(v_n)$ are initialized to the priors $p(u_n = 1) = p(v_n = 1) = 0.01$.

1.8 Algorithmic Complexity

The complexity of ReMixT is determined largely by the amount of sequencing, number of tumour samples, and complexity of the genome. Preprocessing requires calculation of several relationships: a) reads overlapping SNPs, b) reads contained within segments, c) SNPs contained within segments and d) SNPs contained within haplotype blocks. In general calculation of these relationships requires an $\mathcal{O}(n \log n)$ sorting operations on reads, segments and SNPs, followed by $\mathcal{O}(n + m)$ operations to calculate the relationship. Thus in general the preprocessing steps are $\mathcal{O}(n \log n)$ in number of reads, number of SNPs, number of haplotype blocks, and number of segments.

The complexity of the inference method is not dependent on the number of reads, since reads are condensed into read counts per segment. The calculation of the ELBO bounds the overall complexity of one inference step of the variational method, in particular, the summation over c, c' and b, the two sets of copy number states and the breakpoint states. By inspection, the ELBO calcuation performed naively would be $\mathcal{O}(NS^2TM)$ for N segments, S segment copy number states, T breakpoint states, and M clones. Note that the M clones term originates from within the transition factor, since we are calculating a transition cost per clone.

We show in Section 1.5.6 that it is possible to leverage the symmetry of the transition factor to perform the summation of segment and copy number states more efficiently. Specifically, for each clone we can build a table of values for each segment copy number difference as given by Equation 52 using $\mathcal{O}(c_{\max}T)$ operations. We can then use this table in a summation over c and c' requiring $\mathcal{O}(S^2)$ operations. Thus in total the complexity of the ELBO calculation is $\mathcal{O}(NS^2M + Nc_{\max}TM)$.

Two other computationally intensive calculations are the sum/max product algorithms, and the expectation of the transition given the breakpoint variational approximation $q(B)$. The sum and max product algorithms are both $\mathcal{O}(NS^2)$ complexity. Calculation of the transition expectation (Equation 48) again leverages the symmetry of the transition factor (Equation 53), and requires complexity $\mathcal{O}(BS^2M + Bc_{\max}TM)$ for B breakpoints. Since N bounds B, the complexity of the ELBO bounds the complexity of one inference step. The overall complexity of the method is thus $\mathcal{O}(IR(NS^2M + Nc_{\max}TM))$ for I iterations and R restarts.

The memory consumption of the inference method is dominated by the allocation of memory for segment specific transition matrices and pairwise posterior marginals, each requiring $\mathcal{O}(NS^2)$ space. For the current implementation with $S \approx 100$ and $N \approx 20,000$, the algorithm requires 1.6GB each for the transition matrices / marginals at double precision.

Processing of multiple samples occurs in parallel, and thus both preprocessing and inference are linear in the number of samples. One exception is phasing of haplotype blocks within segments, for which all available samples are used to increase phasing accuracy, however, this has no effect on the complexity. Since the segmentation is augmented by breakpoints, the number of segments is dependent on the complexity of the genome. Increased power to detect subclonal breakpoints in a multi-sample setting will increase the number of modelled segments. However, tumours rarely contain 10,000 or more breakpoints, thus although the number of segments may increase with additional samples and additional complexity of the genome, the number of segments will be within the same order of magnitude as for a tumour with few rearrangements.

To summarize, the time and memory complexity of ReMixT are largely dominated by the complexity of the ReMixT model. In particular, the number of clones increases the time complexity directly, and increases both the time and memory complexity as it requires a larger state space for

both segment and breakpoint copy number.

2 Realistic Simulations of Bulk Genome Sequencing

2.1 Simulating Evolutionary Histories

We developed a principled method of simulating rearranged genomes that fulfilled two important criteria. First, the simulated tumour genomes are required to have been produced by a known evolutionary history composed of duplication, deletion, and balanced rearrangement events applied successively to a initially non-rearranged normal genome. Second, the copy number profile of the simulated tumour genome should be reasonably similar to that of previously observed tumours.

Figure 17: (A) Realistic rearranged genomes are simulating using a successive resampling of a fixed population of genomes, similar to the Moran Process. Simulation starts with a set of normal genomes (leftmost column of white circles). At each step, a random rearrangement is applied to produce a new set of genomes (darkening grey circles from left to right), and a new set of genomes is resampled (edges between columns of circles). Two clones are selected from a single evolutionary history (red tinted). (B) Read depth versus GC Percent for 100 kb segments calculated for NA12878 WGS. (C) Read depth versus GC Percent for 100 kb segments for a dataset simulated by resampling NA12878 WGS reads. (D) Example copy number profile obtained using the read resampling method applied to NA12878 WGS reads.

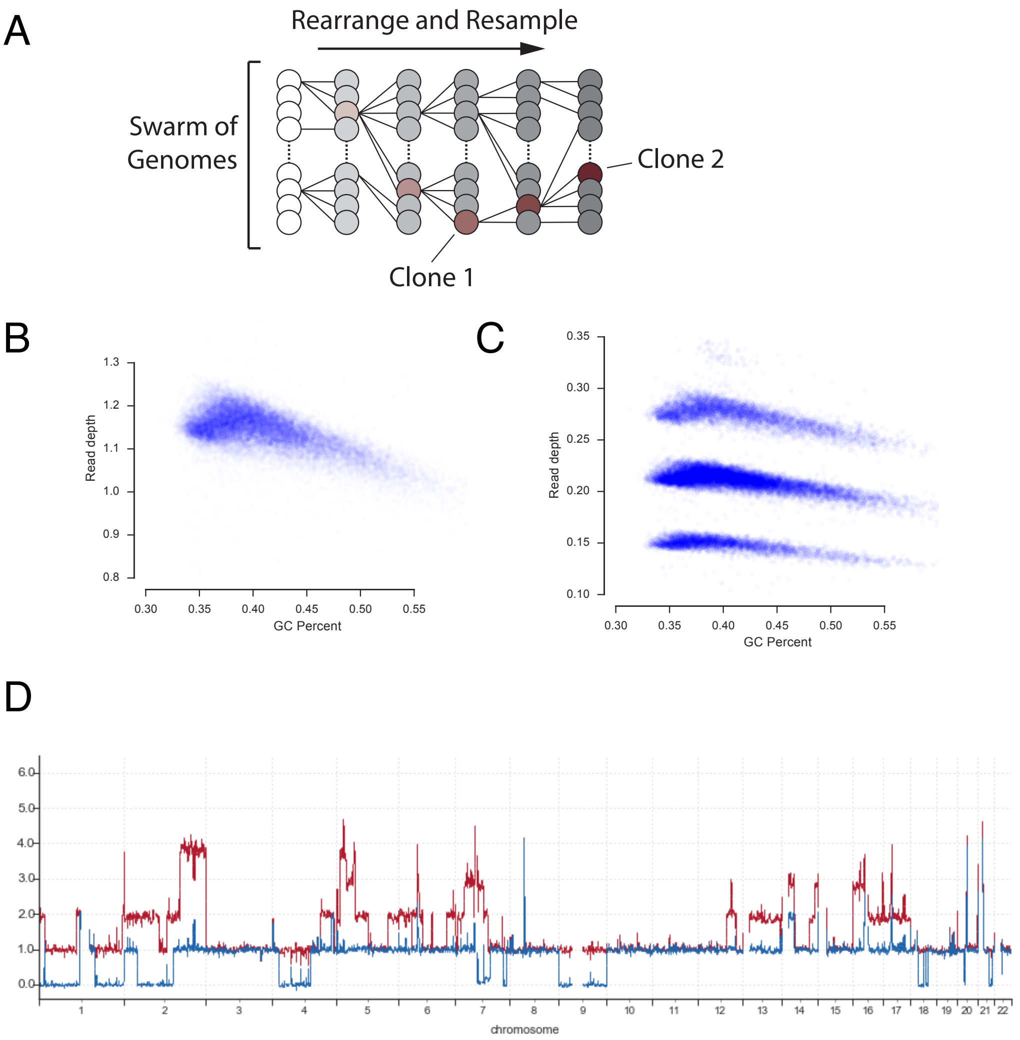

Naively applying random rearrangements to a normal genome would result in a significant number of regions that are homozygously deleted. Such a scenario is unrealistic given that large scale homozygous deletion would remove housekeeping genes necessary for the survival of the cell. Alternatively, a naively simulated genome could become exceptionally large, an outcome that is rarely observed given the presumed burden of replicating such a genome. Thus, we developed a re-sampling method for producing realistic rearranged genomes (Figure 17a). At each step in the simulation of an evolutionary history, we re-sample a swarm of genomes according to a *fitness* function. As a fitness function, we use product of an exponential distribution over the length of homozygous deletions and a gaussian over the ploidy.

To simulate a mixture of related genomes, we first simulate the rearrangement history of the common ancestor. Using a single selected ancestor genome as a starting point, we then simulate additional rearrangements to produce a swarm of potential descendant genomes. Finally we select the descendant genome that is closest to a target deviation between ancestor and descendant. The target deviation is specified as the proportion of the genome with divergent copy number state between ancestor and descendant.

2.2 Simulating genome sequence data

We developed 2 methods for simulating genome sequence data. The first and most simplistic method we call a segment count simulation. From the genome mixture we calculate the expected read count of each segment based on the global haploid read depth, simulated segment copy number, and a randomly sampled proportion of genotypable reads. We then sample read counts from the likelihood model, using parameters learned from the HCC1395 cell line.

The second method, aligned read re-sampling, attempts to generate realistic sequencing data from an existing *source* dataset. The re-sampling method is able to produce data with a realistic GC bias, similar to the GC bias of the source dataset (Figure 17b and 17c). For each read in the source dataset we calculate the probability of sampling that read in a single random draw from all reads, where the probability of sampling a read is proportional to the relative proportion of the containing segment in the simulated genome mixture. Specifically, for read i from the sequencing dataset, let n_i be the segment that contains read i. Calculate $p_{i\ell}$, the probability of selecting read i from allele ℓ in a single random draw, as given by Equation 60. Approximate the normalization constant Z using Equation 61, where x_n is the total number of reads contained within segment n.

$$p_{i\ell} = \frac{1}{Z} \sum_m \rho_m c_{n_i m \ell} \tag{60}$$

$$Z \approx \sum_n x_n \sum_m \rho_m \sum_\ell c_{nm\ell} \tag{61}$$

$$\lambda_{i\ell} = p_{i\ell} * K \tag{62}$$

Next, calculate $\lambda_{i\ell}$, the expected number of copies of read i from allele ℓ in the re-sampled dataset given an expected number of total sampled reads K, as given by Equation 62. Finally, sample $k_{i\ell}$ copies of read i, allele ℓ, from a Poisson distribution with parameter $\lambda_{i\ell}$. Use of the Poisson allows on-line sampling of reads, and is valid given that the act of sampling reads involves sampling many events each with very small probability of occurring. Note that additional noise incurred by re-sampling an existing dataset depends on the size of the source dataset. Thus, we recommend using a source sequencing dataset significantly larger than the target re-sampled dataset to avoid adding additional noise and re-sampling too many duplicate reads.

Simulation of allele specific mismatches at SNP positions requires ground truth assignment of SNP alleles to parental chromosomes. We first simulate parental chromosomes by selecting

recombination positions at a rate of 20 per 100mb, then selecting a random individual from the thousand genomes reference panel for each recombined segment, and combining the chromosomes of the resulting individuals into a single simulated individual. Fully resolved parental chromosomes of a well studied individual could also be used, though for the described method, parental chromosomes of the source dataset are not required, as the allele specific measurements of the source dataset are not used in the simulation. Instead, SNPs contained within the start and end positions of re-sampled reads are assigned the genotype of the simulated chromosome given the ground truth parental chromosome data. A proportion of SNPs are then modified at random according to a configurable base call error. An example copy number profile obtained using the re-sampling method is shown in Figure 17d.

2.3 Simulation parameters for breakpoint evaluation

We performed two sets of simulations, varying the proportion of subclonal genomic segments in set A, and the descendant clone fraction in set B. All simulations involved a normal clone at 40%, and 2 tumour clones at varying proportions making up the remaining 60%. For set A we simulated 40 genome mixtures, with 10 each having proportion of subclonal genomic segments 15%, 30%, and 45%. Descendant clone fraction for set A was fixed at 40%. For set B we simulated 50 genome mixtures, with 10 each having descendant clone fractions 5%, 15%, 30%, 45%, and 55%. Proportion of subclonal genomic segments for set B was fixed at 30%. Each genome was simulated to have 100 rearrangements in the ancestral genome, and 50 additional rearrangements in a descendant genome. Additionally, we simulated a noisy breakpoint prediction process by adding an additional 50 false breakpoints and removing 10% of the real breakpoints from the set of breakpoints provided to the method.

We assumed 20X sequencing coverage [5]. Read counts were simulated using a mixture of 2 negative binomial distributions with over-dispersion parameter $r = 1000$ and $r = 10$ respectively, and mixing fraction 0.01 for the second higher variance (lower over-dispersion) component. Proportion genotypable reads for each segment was sampled uniformly from between 0.05 and 0.2. Allele read counts were sampled from a mixture of 2 beta binomial distributions with over-dispersion parameter $M = 2000$ and $M = 10$ respectively, and mixing fraction 0.01 for the second higher variance (lower over-dispersion) component.

2.4 Simulation parameters for comparison with existing methods

We simulated 2 sets of genome mixtures as described in Section 2.3, with each set consisting of 4 genome mixtures per varied parameter instead of 10 as for the segment simulation. As a source dataset we used a 200X genome sequencing dataset provided by illumina [5] for the NA12878 hapmap individual. Sequence files were downloaded from `http://sra.dnanexus.com` (accession ERP001775), and aligned using bwa 0.7.12 [9]. We sampled reads from this dataset as described in Section 2.2 based on an approximate coverage of 30X.

2.5 Post-hoc calculation of breakpoint copy number

For the HMM model, we calculate breakpoint copy number post-hoc using a simple greedy algorithm. We iterate through the list of predicted breakpoints and at each step, attempt to increase

[5] total haploid coverage of 0.1 reads (paired) per nucleotide corresponds to a 20X sequence coverage genome, 100X100bp reads from ∼300bp fragments

the breakpoint copy number if doing so would explain a transition in copy number at each breakend. The algorithm stops when the copy number cannot be increased on any breakpoint.

3 Supplementary Analysis

3.1 Evaluation of Outlier Estimation

We evaluated the ability of ReMixT to infer the correct outlier state of each segment using the segment count simulations. For each segment of each simulation, we compared the simulated outlier state of the likelihood to the inferred state using ReMixT, distinguishing between the outlier state of the total and allele likelihoods. Most important was to confirm that segments called as outliers by ReMixT are in fact true simulated outliers. Thus for each simulation we calculated the precision of outlier inference for both total and allele specific outlier states. A segment with outlier probability of 0.5 or greater by ReMixT was considered to have been inferred as an outlier by ReMixT. In this context, we defined precision as the number of true outlier segments predicted by ReMixT as a proportion of the total number of predicted outlier segments. Precision for outlier inference was in the range of 0.8-0.99 and decreased with increasing proportion of subclonal segments (Figure 18).

Figure 18: Precision of outlier state inference by ReMixT for segment count simulations. Shown in the first row is the outlier precision for the total read count likelihood varying (A) descendant clone fraction and (B) proportion of the genome subclonal. Shown in the first row is the outlier precision for the allele read count likelihood varying (C) descendant clone fraction and (D) proportion of the genome subclonal. Bars depict mean precision and vertical lines the 95% confidence interval.

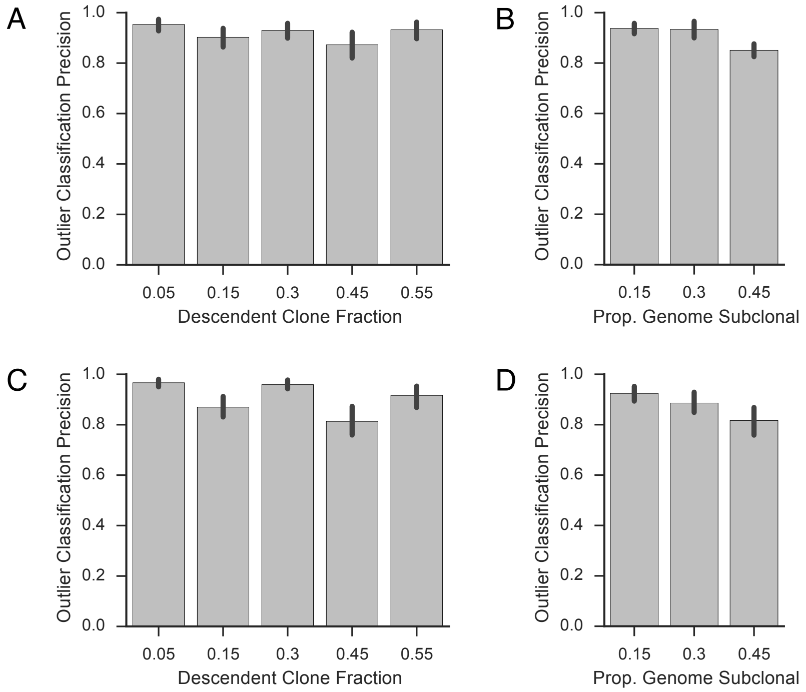

3.2 Evaluation of Performance for 3 Tumour Clone Mixture

We evaluated the performance of ReMixT when applied to simulated mixtures of 3 tumour clones and a contaminating normal clone. The parameters of the simulation were identical to the those of the breakpoint evaluation (Section 2.3), with the exception of an additional simulated tumour clone. As described Section 2.1, we first generated an ancestral tumour clone by successively rearranging a wild type genome, then generated a descendant genome by successively rearranging the ancestral genome. For the 3 tumour clone mixture, we generated a second descendant genome by again successively rearranging the ancestral genome. The normal and ancestral clone fractions were set as before to a specific set of values as described in Section 2.3. The fractions of the two descendant clones were randomly generated to sum to the remaining fraction of the mixture.

ReMixT models a mixture of 2 tumour clones. As expected, ReMixT performed significantly worse on the simulated 3 tumour clone mixtures than on the 2 tumour clone mixtures described in Section 2.3 (Figure 19). An exception was for the mixture of 2 tumour clones at equal proportions for which ReMixT called dominant copy number correctly for a lower than average proportion of the genome (Figure 19a). For one of the simulations, the ReMixT copy number and normal fraction were categorically incorrect (Figures 19b and 19d). For the remaining simulations, normal fraction and dominant copy number calls were variable, and ReMixT produced reasonable solutions for a subset of simulations especially when the two descendant tumour clones together represented a small fraction of the mixture. Precision of breakpoint presence/absence prediction was marginally lower than for the 2 tumour clone mixtures, whereas recall was significantly worse, reflecting the difficulty of identifying lower prevalence breakpoints in more heterogeneous tumours (Figures 19e-19h). Precision and recall of breakpoint subclonality showed a similar trend.

Figure 19: Simulation results for ReMixT on 3 and 4 clone mixtures (1 normal clone, and 2 and 3 tumour clones). Two sets of simulations were performed, varying fraction of the descendant tumour clone (left column) and proportion of the genome with divergent copy number (right column). Boxplots show proportion of the genome for which the tool correctly called dominant copy number (A, B), relative normal fraction error (C, D), and precision (E, F) and recall (G, H) of breakpoint presence absence classification. Boxes show the interquartile (IQR) range with a line depicting the median. Whiskers extend 1.5 × IQR above quartile 3 and below quartile 1. Diamonds show positions of outlier data points.

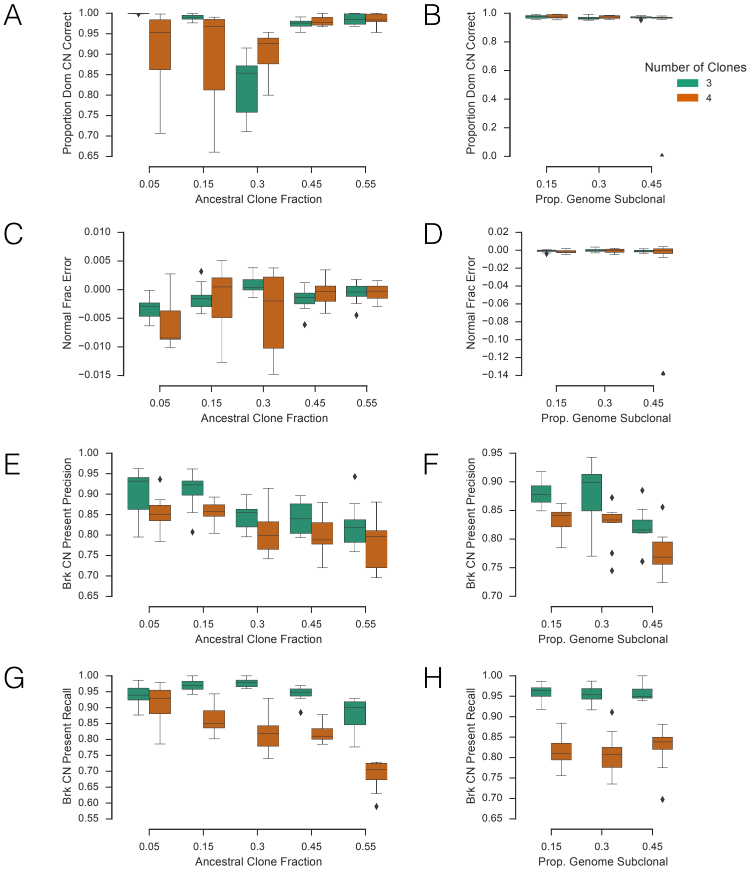

3.3 Evaluation of Performance with Increased Sequencing Depth

We evaluated the performance of ReMixT when applied to simulated mixtures sequenced to 20X and 40X depth. The parameters of the simulation were identical to the those of the breakpoint evaluation (Section 2.3). Matched genome mixtures with equivalent evolutionary histories and clonal fractions differed only by simulated sequencing depth. The ReMixT results for the 40X datasets were similar to the 20X datasets, with no significant difference in performance metrics identified by paired t-test.

Figure 20: Simulation results for 20X and 40X sequencing coverage. Two sets of simulations were performed, varying fraction of the descendant tumour clone (left column) and proportion of the genome with divergent copy number (right column). Boxplots show proportion of the genome (A, B), and proportion of breakpoints (C, D) for which the tool correctly called clone specific copy number, in addition to relative normal fraction error (E, F), and relative minor clone fraction error (G, H). Boxes show the interquartile (IQR) range with a line depicting the median. Whiskers extend 1.5 $\times$ IQR above quartile 3 and below quartile 1. Diamonds show positions of outlier data points.

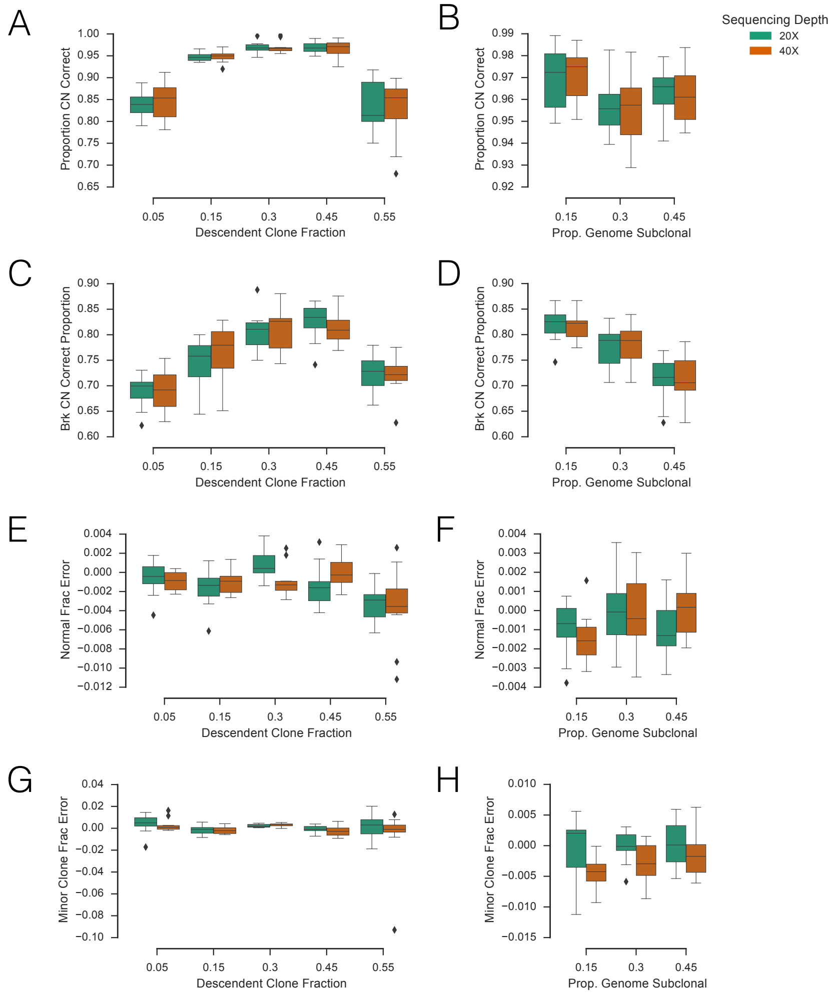

3.4 Comparison of CPU Time and Memory Consumption

We tracked the CPU time used by each method when applied to the simulated datasets for the comparison benchmarking. The CPUs used for the analysis were Intel(R) Xeon(R) CPU E5-4640 v2 @ 2.20GHz with 500GB memory. ReMixT required the highest CPU time of all the methods with Battenberg a close second, and CloneHD requiring the least amount of time (Figure 21a). Required CPU time paralleled overall performance, as Battenberg performed best of the competing methods and CloneHD performed poorly on many metrics. A significant amount of time spent by

ReMixt could be attributed to inferring haplotype blocks (Figure 21b). We anticipate that future improvements in haplotype block inference could significantly reduce the CPU time required for ReMixT (and also Battenberg). Furthermore, establishment of haplotype inference as a standard analysis and prerequisite for other analyses would reduce the marginal cost of running ReMixT.

Figure 21: Total CPU time in seconds required for each tool (A) and each step in the ReMixT method (B). Bars depict mean CPU times across simulated datasets and horizontal lines the 95% confidence interval.

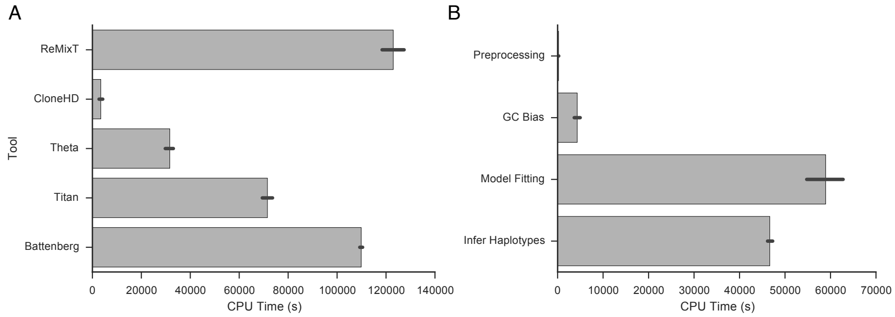

Maximum allowable memory was increased incrementally for each tool until memory errors were rare. Memory requirements varied from 8-32GB per process (Table 2).

Table 2: Maximum allowable memory consumption for each tool.

Tool Name	Memory (GB)
ReMixT	20
CloneHD	8
THetA	32
TITAN	16
Battenberg	8

3.5 Comparison with existing methods for SA501X3F

We compared ReMixT with CloneHD, TITAN, and THetA using SA501X3F bulk and matched single cell sequencing [13]. Single cell data was analyzed using previously described techniques [13, 6]. Three clones were identified at proportions 0.82, 0.11, 0.07. Clones B and C were similar and formed a distinct clade. Thus to facilitate benchmarking with the number of tumour clones fixed at 2, we merged clones B, C and herein refer to the combined clone as clone B in subsequent analyses.

Next we used ReMixT with CloneHD, TITAN, and THetA to predict clone specific total copy number using matched bulk whole genome sequencing, comparing the results with the ground-truth obtained from the single cell sequencing (Figure 22). ReMixT, TITAN and THetA were each able to estimate minor clone fraction (clone B proportion) with reasonable accuracy (Figure 22i). ReMixT was able to recover the copy number of the dominant clone (clone A) with high accuracy (Figure 22a,22b), whereas accuracy with respect to clone A and B together was highest for segments in lower copy number states (≤ 3 total copies). CloneHD performed poorly, largely as a result of over-fitting with a solution consisting of two highly divergent clones (Figure 22c,22d,22j). TITAN was able to recover the copy number of the dominant clone with reasonable accuracy for lower copy

clone A copy number (Figure 22g). However, TITAN was in general too conservative with respect to subclonal regions, predicting many truly subclonal regions as clonal, resulting in low accuracy with respect to the copy number of both clones (Figure 22h). THetA was able to recover the copy number of the dominant clone with high accuracy (Figure 22e), but accuracy with respect to the copy number of both clones was variable (Figure 22f).

Figure 22: Performance comparison of ReMixT with CloneHD, TITAN, and THetA using SA501X3F bulk and matched single cell sequencing. Each plot shows the proportion of genomic length for which the tool correctly called copy number of the dominant clone A (y-axis, left column), and copy number of both clone A and B (y-axis, right column), versus the copy number of clone A (x-axis). Plots show the results for ReMixT (A, B), CloneHD (C, D), TITAN (E, F) and THetA (G, H). Also shown is the minor clone fraction (I) and proportion of the genome predicted to have subclonal copy number (J) as predicted by each method.

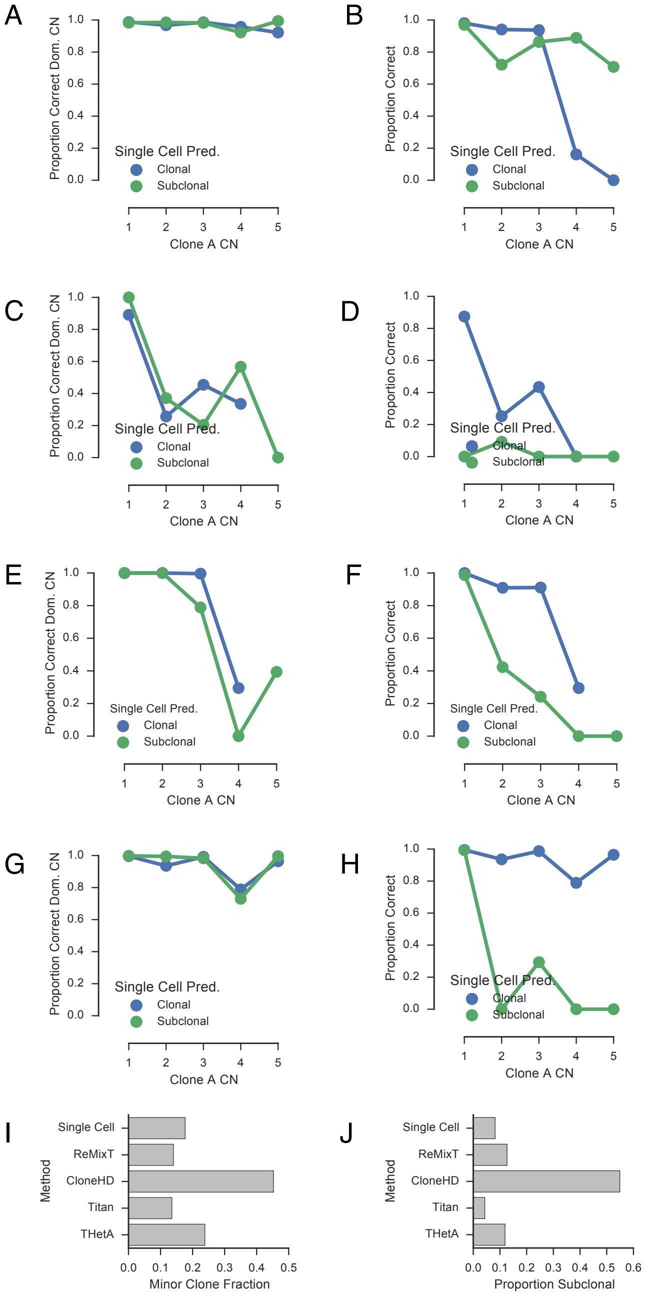

4 Parameters used for existing methods

4.1 THetA2.0

We ran BICSeq2 with default parameters on input tumour and normal read counts to generate an input segmentation for THetA. We then ran THetA using the RunTHetA program, adding the `--FORCE` argument to force THetA to run on genomes which were sub-optimal candidates for THetA analysis. We used the solution with 2 tumour clones by default unless specified in the analysis. Note that in some instances, THetA failed to complete when using allele information, and in this situation we fall back to running THetA without allelic counts. When running THetA either with and without allelic counts, a time limit of 48 hours is set, after which THetA analysis is interrupted, and THetA is assumed to have failed.

4.2 TITAN

As input to TITAN, we calculated counts of reads contained within regular 1000bp segments, and counts of reads supporting the reference and non-reference allele for heterozygous germline SNPs. We used multiple initializations, with normal contamination from 0 to 1 in increments of 0.1, and ploidy from 1 to 4 in increments of 1. The number of TITAN clusters was fixed at 2. We then selected the solution with lowest `S_Dbw validity index`. Since the parameterization for TITAN is slightly different than for other tools, we used the following formula to convert from estimates of tumour clone prevalences t_1 and t_2, and normal contamination estimate n, as follows.

$$\text{mixture} = [n, (1-n) \times t_2, (1-n) \times |t_1 - t_2|] \tag{63}$$

4.3 CloneHD

As input to CloneHD, we calculated counts of reads contained within regular 1000bp segments, and counts of reads supporting the reference and non-reference allele for heterozygous germline SNPs (b-allele frequency (BAF) data). We used a series of steps as outlined in `run_example.sh`.

1. use filterhd to analyze the normal read depth data for technical read depth modulation
2. use filterhd to analyze the tumour read depth data to get a benchmark of the log likelihood
3. use filterhd to analyze the tumour read depth data with the bias estimate from the normal
4. use filterhd to analyze the tumour BAF data
5. use clonehd to infer copy number based on tumour read depth and BAF data.

4.4 Battenberg

Battenberg version 1.5.3 was installed from source from github `http://github.com/cancerit/cgpBattenberg`. As input, we calculated reference and alternate read counts for 1000 genomes SNPs and provided these as direct input to the `battenberg.pl` script (`-allele-counts` option). For the comparison simulations, chromosome X was removed from the Battenberg configuration files to facilitate analysis of simulated chromosomes 1 through 22. Output VCFs were parsed, with one non-standard adjustment. Although the `TCN` field is annotated as *Total copy number* in the VCF header, the results were only accurate if this field was interpreted as *Major copy number*.

References

[1] Yuval Benjamini and Terence P Speed. Summarizing and correcting the gc content bias in high-throughput sequencing. *Nucleic Acids Res*, 40(10):e72, May 2012.

[2] C Bishop. Pattern recognition and machine learning (information science and statistics), 1st edn. 2006. corr. 2nd printing edn. *Springer, New York*, 2007.

[3] Richard H Byrd, Peihuang Lu, Jorge Nocedal, and Ciyou Zhu. A limited memory algorithm for bound constrained optimization. *SIAM Journal on Scientific Computing*, 16(5):1190–1208, 1995.

[4] Olivier Delaneau, Jonathan Marchini, and Jean-François Zagury. A linear complexity phasing method for thousands of genomes. *Nat Methods*, 9(2):179–81, Feb 2012.

[5] Michael A Eberle, Epameinondas Fritzilas, Peter Krusche, Morten Källberg, Benjamin L Moore, Mitchell A Bekritsky, Zamin Iqbal, Han-Yu Chuang, Sean J Humphray, Aaron L Halpert, et al. A reference dataset of 5.4 million human variants validated by genetic inheritance from sequencing a three-generation 17-member pedigree. *bioRxiv*, page 055541, 2016.

[6] Peter Eirew, Adi Steif, Jaswinder Khattra, Gavin Ha, Damian Yap, Hossein Farahani, Karen Gelmon, Stephen Chia, Colin Mar, Adrian Wan, Emma Laks, Justina Biele, Karey Shumansky, Jamie Rosner, Andrew McPherson, Cydney Nielsen, Andrew J L Roth, Calvin Lefebvre, Ali Bashashati, Camila de Souza, Celia Siu, Radhouane Aniba, Jazmine Brimhall, Arusha Oloumi, Tomo Osako, Alejandra Bruna, Jose L Sandoval, Teresa Algara, Wendy Greenwood, Kaston Leung, Hongwei Cheng, Hui Xue, Yuzhuo Wang, Dong Lin, Andrew J Mungall, Richard Moore, Yongjun Zhao, Julie Lorette, Long Nguyen, David Huntsman, Connie J Eaves, Carl Hansen, Marco A Marra, Carlos Caldas, Sohrab P Shah, and Samuel Aparicio. Dynamics of genomic clones in breast cancer patient xenografts at single-cell resolution. *Nature*, 518(7539):422–6, Feb 2015.

[7] Andrej Fischer, Ignacio Vázquez-García, Christopher J R Illingworth, and Ville Mustonen. High-definition reconstruction of clonal composition in cancer. *Cell Rep*, 7(5):1740–52, Jun 2014.

[8] Gavin Ha, Andrew Roth, Jaswinder Khattra, Julie Ho, Damian Yap, Leah M Prentice, Nataliya Melnyk, Andrew McPherson, Ali Bashashati, Emma Laks, Justina Biele, Jiarui Ding, Alan Le, Jamie Rosner, Karey Shumansky, Marco A Marra, C Blake Gilks, David G Huntsman, Jessica N McAlpine, Samuel Aparicio, and Sohrab P Shah. Titan: inference of copy number architectures in clonal cell populations from tumor whole-genome sequence data. *Genome Res*, 24(11):1881–93, Nov 2014.

[9] Heng Li and Richard Durbin. Fast and accurate long-read alignment with burrows-wheeler transform. *Bioinformatics*, 26(5):589–95, Mar 2010.

[10] Layla Oesper, Ahmad Mahmoody, and Benjamin J Raphael. Theta: inferring intra-tumor heterogeneity from high-throughput dna sequencing data. *Genome Biol*, 14(7):R80, 2013.

[11] Adam B Olshen, ES Venkatraman, Robert Lucito, and Michael Wigler. Circular binary segmentation for the analysis of array-based dna copy number data. *Biostatistics*, 5(4):557–572, 2004.

[12] Christopher Yau. Oncosnp-seq: a statistical approach for the identification of somatic copy number alterations from next-generation sequencing of cancer genomes. *Bioinformatics*, 29(19):2482–4, Oct 2013.

[13] Hans Zahn, Adi Steif, Emma Laks, Peter Eirew, Michael VanInsberghe, Sohrab P Shah, Samuel Aparicio, and Carl L Hansen. Scalable whole-genome single-cell library preparation without preamplification. *Nat Methods*, 14(2):167–173, Feb 2017.
